# Abundant Yet Aberrant T Helper Cell Responses to *Candida albicans* Underlie Mucosal Candidiasis in Humans and Mice

**DOI:** 10.1002/eji.70065

**Published:** 2025-10-06

**Authors:** Camilla Basso, Corinne De Gregorio, Roberta Marzi, Florian Kirchner, Gabor Gyülveszi, Mélanie Migaud, Sinu Paul, Alessandro Sette, Antonio Lanzavecchia, Salomé LeibundGut‐Landmann, Jean‐Laurent Casanova, Anne Puel, Simone Becattini, Federica Sallusto

**Affiliations:** ^1^ Institute for Research in Biomedicine Università della Svizzera italiana Bellinzona Switzerland; ^2^ Section of Immunology Vetsuisse Faculty and Institute of Experimental Medicine University of Zürich Zürich Switzerland; ^3^ Laboratory of Human Genetics of Infectious Diseases Necker Branch INSERM U1163 Paris France; ^4^ Paris Cité University Imagine Institute Paris France; ^5^ La Jolla Institute for Immunology La Jolla California USA; ^6^ National Institute of Molecular Genetics Milan Italy; ^7^ Medical Research Council Centre for Medical Mycology at the University of Exeter Department of Biosciences Faculty of Health and Life Sciences Exeter UK; ^8^ St. Giles Laboratory of Human Genetics of Infectious Diseases Rockefeller Branch Rockefeller University New York NY USA; ^9^ Department of Pediatrics Necker Hospital for Sick Children Paris France; ^10^ Howard Hughes Medical Institute New York NY USA; ^11^ Department of Pathology and Immunology University of Geneva Geneva Switzerland; ^12^ Geneva Centre for Inflammation Research University of Geneva Geneva Switzerland; ^13^ Institute of Microbiology ETH Zurich Zurich Switzerland

**Keywords:** CD4 T cells, cytokines, host/pathogens interactions

## Abstract

T helper cell subsets—Th1, Th2, and Th17—coordinate pathogen‐specific immune responses. *Candida albicans*‐specific T cells include protective Th17 cells alongside other Th subsets. However, the role of alternative Th subsets remains unclear, particularly in individuals with impaired Th17 responses and recurrent candidiasis. Here, we show that patients with STAT1 gain‐of‐function mutations and chronic mucocutaneous candidiasis have a numerically normal but functionally altered pool of *C. albicans*‐specific Th cells, skewed toward Th1 and Th2. This imbalance persisted even when assessing responses to the known and the newly identified immunodominant *C. albicans* antigens MP65 (65‐kilodalton mannoprotein), HYR1 (hyphally regulated cell wall protein 1), and SAP4‐6 (secreted aspartic proteinases 4–6), suggesting that antigen recognition and priming remain intact despite qualitative defects in T cell polarization. Using mucosal infection mouse models, we demonstrate that *C. albicans*‐specific transgenic Th17 cells are sufficient to control infection, whereas Th1 and Th2 cells fail to protect, even in high numbers. Moreover, co‐transfer of Th2 cells with Th17 cells impaired fungal control via an IL‐4‐dependent mechanism. These findings highlight the essential role of Th17 cells in protective immunity against *C. albicans* and reveal that non‐Th17 responses are ineffective and may contribute to susceptibility in both humans and mice.

## Graphical abstract

Th17 cells confer protection against *Candida albicans* infection. In patients with STAT1 gain‐of‐function (GOF) mutations and chronic mucocutaneous candidiasis (CMC), Candida‐specific Th cell responses are numerically preserved but skewed toward Th1 and Th2. In a mouse model of mucosal vaginal infection, Th1 and Th2 cells fail to control fungal growth, and Th2 cells can even impair Th17‐mediated protection. Image created with BioRender.

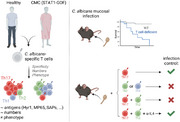

## 1 Introduction

*Candida albicans* is a fungal species that typically resides as a commensal on mucosal surfaces, including the oral cavity, gastrointestinal tract, skin, and vagina [1]. While it is generally harmless as part of the microbiota, *C. albicans* can become a pathogen, leading to superficial diseases affecting the skin, nails, scalp, and/or mucosa, such as vulvovaginal candidiasis (VVC) in women of reproductive age, oropharyngeal candidiasis (OPC), or chronic mucocutaneous candidiasis (CMC) in immunocompromised individuals [2, 3, 4, 5]. These include patients with acquired (HIV, or those undergoing chemotherapy or posttransplant treatment [6]) or primary immunodeficiencies (PIDs or inborn errors of immunity [IEIs] [7]), as well as patients without any deficit identified yet. *C. albicans* can also disseminate through the bloodstream, causing life‐threatening invasive diseases [8].

Clinical and experimental evidence underscores the importance of Th17‐mediated immune responses in protecting against *C. albicans* infections. In PIDs, mutations of genes essential for type‐17 immunity underlie CMC [9, 10]. Additionally, polymorphisms in genes regulating type‐17 immunity have been linked to recurrent VVC [11, 12]. The protective role of Th17 cells is further highlighted by increased candidiasis rates in patients receiving IL‐17 inhibitors [13]. In murine models, deficiencies in IL‐17 pathway components, such as IL‐23, IL‐17 receptor, or the adaptor protein Act1, lead to susceptibility to oral and cutaneous candidiasis [14, 15, 16, 17, 18, 19]. Similarly, neutralizing IL‐17A compromises immunity in models of oral mucosal infection [15, 20].

In prior studies, we demonstrated that memory CD4^+^ T cells responding to *C. albicans* in healthy individuals are functionally diverse, comprising Th17 cells, IFN‐γ‐producing CCR6^+^ Th1* cells and, at lower frequency, Th1 and Th2 cells [21]. However, the role of IFN‐γ‐producing Th1 cells and IL‐4‐producing Th2 cells in either protection against or pathogenesis of *C. albicans* infection remains unclear. In the present study, we first investigated Th cells in CMC patients harboring heterozygous STAT1 gain‐of‐function (GOF) variants that cause immune dysregulation characterized predominantly by recurrent bacterial, viral, and fungal diseases, with the latter affecting almost all patients in the form of chronic oropharyngeal, cutaneous, esophageal, genital, and/or onychomycoses [22]. We found that in these patients, the response *to C. albicans* is dominated by Th1 and Th2 cells, while Th17 cells are markedly reduced. To investigate these findings further, we developed an in vivo model of VVC using *Cd3e^–/–^* mice reconstituted with *C. albicans*‐specific TCR‐transgenic T cells polarized toward distinct Th phenotypes [23]. Our results demonstrate that only IL‐17A‐producing Th17 cells were effective in clearing *C. albicans* from mucosal tissues, whereas Th1 and Th2 cells failed to provide protection, and Th2 could even impair Th17‐mediated protection.

## 2 Results

### 2.1 STAT1‐GOF Patients with CMC Display a Quantitatively Preserved, but Phenotypically Altered *C. albicans*‐specific Th Response

**FIGURE 1:**
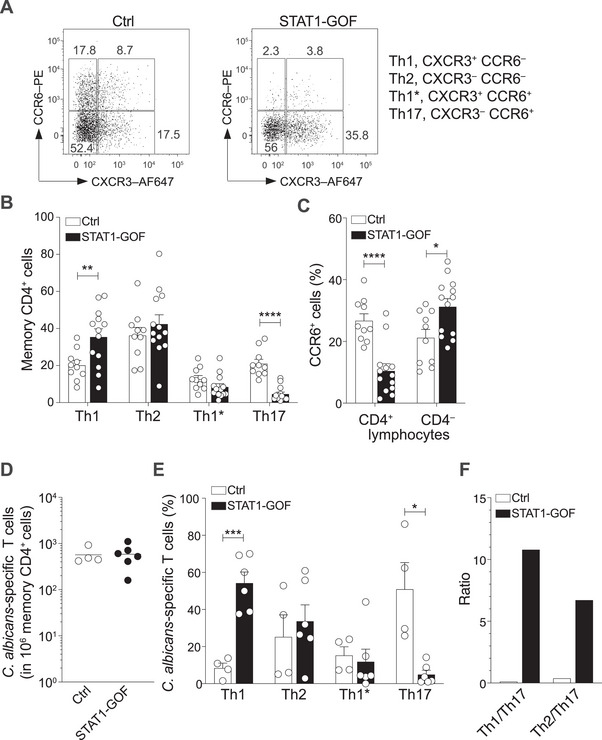
Frequency and distribution of *C. albicans*‐specific memory T cells in STAT1‐GOF patients with CMC. (A) CCR6 and CXCR3 expression in memory CD4^+^ T cells pregated as Lin^–^CD4^+^CD45RA^–^ in a representative healthy control (Ctrl) and a representative STAT1‐GOF patient. Shown in the plots are the percentages of Th1 (CXCR3^+^CCR6^–^), Th2 (CXCR3^–^CCR6^–^), Th1* (CXCR3^+^CCR6^+^), and Th17 (CXCR3^–^CCR6^+^) cells. AF647 = Alexa Fluor 647. (B) Quantification of memory CD4^+^ T cell subsets defined as in (A) from healthy controls (*n* = 10) and STAT1‐GOF patients (*n* = 13). (C) Quantification of CCR6 expression in memory CD4^+^ and CD4^–^ lymphocytes in the same donors. (D) Frequency of circulating *C. albicans*‐specific CD4^+^ T cells in total memory and in memory cell subsets estimated by the T cell library assay in healthy controls (*n* = 4) and STAT1‐GOF patients (*n* = 6). Lines represent the median. (E) Distribution of *C. albicans*‐specific CD4^+^ T cells in Th1, Th2, Th1*, and Th17 subsets, defined as in (A). Values were calculated considering the percentage of each subset within memory CD4^+^ T cells in PBMCs. (F) Ratio between the mean percentages of *C. albicans*‐specific Th1 and Th17 and Th2 and Th17 cells in healthy controls and STAT1‐GOF patients, as shown in (E). In B, C and E, F, values represent mean ± SEM. Statistical significance was determined by unpaired parametric *t*‐test (with Welch's correction) *p < 0.05, **p < 0.01, ***p < 0.001, ****p < 0.0001.

Peripheral blood mononuclear cells (PBMCs) were isolated from STAT1‐GOF patients and from healthy individuals as controls (Table S1). Memory CD4^+^ CD45RA^–^ Th cell subsets were identified based on chemokine receptor expression: Th1 (CXCR3^+^CCR6^–^), Th2 (CCR4^+^CCR6^–^), Th1* (CXCR3^+^CCR6^+^), and Th17 (CCR4^+^CCR6^+^), as previously described [24, 25] (Figure 1A; Figure S1A,B). Quantitative analysis revealed significant shifts in Th subsets among STAT1‐GOF patients, with approximately twice as many Th1 cells and less than half the Th17 cells compared with controls (Figure 1B). CD4^+^ T cells from STAT1‐GOF patients exhibited markedly reduced CCR6 expression, a phenomenon not observed in other leukocyte types (Figure 1C), suggesting distinct regulatory mechanisms for CCR6 expression in specific immune compartments. Furthermore, individual Th17 clones from STAT1‐GOF patients generated by limiting dilution from sorted CCR6^+^CXCR3^–^ memory Th17 cells displayed a significantly higher capacity to produce IFN‐γ compared with Th17 clones from healthy individuals (Figure S2A), consistent with increased STAT1 signaling, although they were not significantly impaired in their capacity to produce IL‐17 and IL‐22.

To determine the frequency of *C. albicans*‐specific cells within each Th subset, we utilized the “T cell library” assay [26], a high‐throughput cellular screen to measure antigen‐specific T cell responses. The frequency of CD4^+^ memory T cells responding to monocytes pulsed with heat‐killed *C. albicans* yeast cells was comparable between patients and healthy individuals (Figure 1D), indicating that the overall magnitude of the T cell response is preserved in STAT1‐GOF patients. However, the distribution of *C. albicans*‐specific T cells in distinct memory subsets differed dramatically (Figure 1E; Figure S2B). In healthy individuals, the response was predominantly Th17, whereas in STAT1‐GOF patients, it was primarily Th1 and Th2. This shift resulted in highly skewed Th1/Th17 and Th2/Th17 ratios in STAT1‐GOF patients (Figure 1F), reflecting a robust yet imbalanced *C. albicans*‐specific response.

### 2.2 Skewed Immune Responses to Immunodominant *C. albicans* Antigens in STAT1‐GOF Patients

**FIGURE 2:**
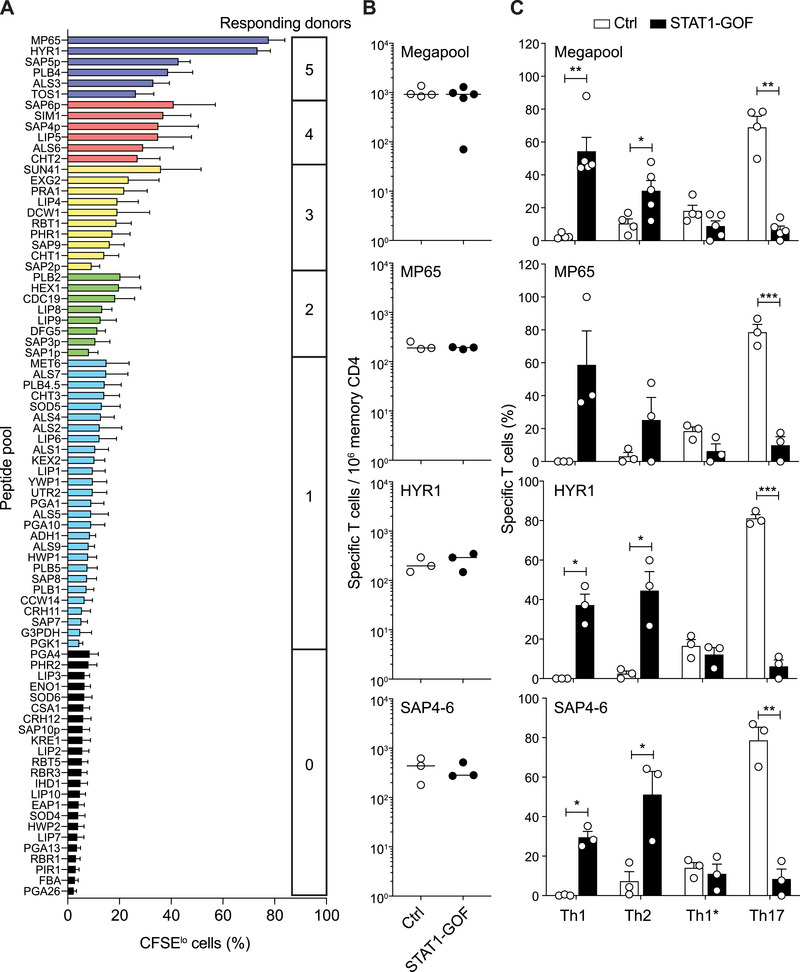
Identification of immunodominant *C. albicans* antigens and frequencies and distribution of memory CD4^+^ T cells specific for immunodominant antigens in STAT1‐GOF patients. (A) Proliferation of memory CD4^+^ T cells in response to peptide pools covering 80 *C. albicans* antigens. CD4^+^CD45RO^+^ memory T cells were isolated from healthy donors (*n* = 5), CFSE‐labeled, and co‐cultured with autologous monocytes pulsed with the individual peptide pools. After 7 days, 50 U/mL IL‐2 was added to the culture, and CFSE dilution was assessed at day 12. Each pool was tested in duplicate for each donor; bars show the percentage (mean + SEM) of proliferating (CFSE^lo^) cells. The number on the right indicates the number of donors in which a significant response (defined as % CFSE^lo^ >5‐times higher than the background proliferation in the absence of antigen) was detected. (B) Frequency of memory CD4^+^ T cells specific for *C. albicans* megapool, MP65, HYR1, or SAP4‐6 antigens, as estimated by the T cell library assay. Each dot represents one donor (*n* = 3 or 5). Lines are median. (C) Distribution of memory CD4^+^ T cells specific for the indicated antigens in different Th subsets; values were calculated considering the percentage of each subset within memory CD4^+^ T cells in PBMCs. Values represent mean + SEM. Statistical significance was determined by an unpaired parametric *t*‐test (with Welch's correction). *p < 0.05, **p < 0.01, ***p < 0.001.

To investigate whether the skewed Th1 and Th2 responses in STA1‐GOF patients result from the recognition of distinct antigens, we first generated an antigenic “fingerprint” of *C. albicans* using T cells from healthy individuals. Based on an extensive literature review, we selected 80 *C. albicans* proteins known to be abundant and either immunogenic or relevant to pathogenicity or fungal biology (Table S2). Peptides predicted to bind common MHC‐II molecules were identified, synthesized as 15‐mers (4–51 peptides per protein), and pooled to recreate the immunogenic core of each protein. Circulating memory CD4^+^ T cells were labeled with CFSE and cultured with autologous monocytes pulsed with peptide pools. Proliferation was measured after 12 days, and antigens were ranked by the fraction of individuals showing detectable proliferation and by the response magnitude (Figure 2A). The top 15 peptide pools, which induced >20% proliferation in at least 80% of individuals, were pooled to form a megapool and used, together with peptides covering the immunodominant antigens MP65, HYR1, and SAP4, SAP5 and SAP6 (SAP4‐6), to quantify the response of memory Th1, Th2, Th1*, and Th17 cells from STAT1‐GOF patients and healthy individuals. Consistent with our earlier data using *C. albicans* cells, T cell library assays revealed comparable frequencies of memory CD4^+^ T cells responding to the megapool in patients and controls (Figure 2B). Within megapool‐specific T cells, the subset distribution was inverted, with a significantly higher Th1 and Th2 response and lower Th17 response in STAT1‐GOF patients compared with controls (Figure 2C). The responses to the immunodominant antigens MP65, HYR1, and SAP4‐6, which collectively accounted for the majority of *C. albicans*‐specific T cells, were of comparable magnitude in patients and controls (Figure 2B). Also in this case, the subset distribution was inverted. In controls, responses were primarily of Th17‐ and Th1*‐type (Th17 + Th1* >> Th2 + Th1). In STAT1‐GOF patients, responses were primarily of Th1‐ and Th2‐type (Th1 + Th2 >> Th1* + Th17) (Figure 2C).

In summary, while the *C. albicans*‐specific Th response in STAT1‐GOF patients is quantitatively preserved and directed against a similar antigen repertoire as in healthy individuals, it is qualitatively altered, with significantly reduced Th17 responses and higher Th1 and Th2 responses.

### 2.3 T Cell Immunity Is Essential for Long‐Term Protection in a Mouse Model of Mucosal Candidiasis

**FIGURE 3:**
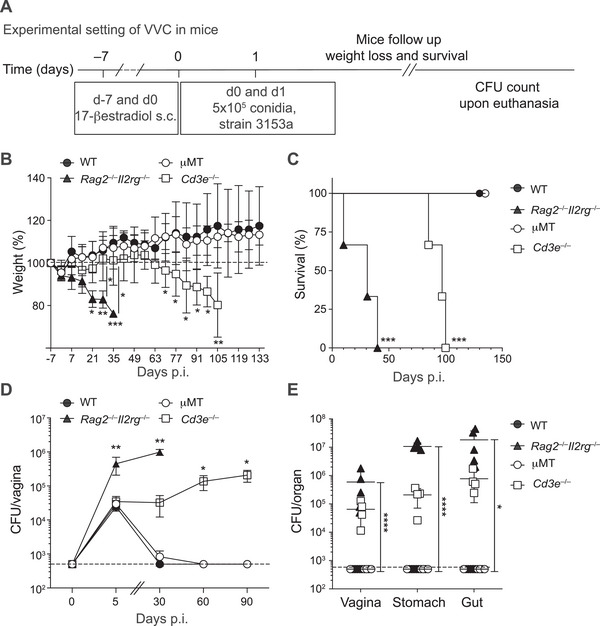
Long‐term protection against *C. albicans* vaginal infection requires T cell immunity. (A) Schematic outline of the experimental approach. WT (*n* = 5), *Rag2* ^–/–^ *Il2rg* ^–/–^ (*n* = 5), µMT (*n* = 5), and *Cd3e* ^–/–^ (*n* = 5) mice were treated with 17‐β‐estradiol and infected with *C. albicans*. Body weight was monitored at different time points. WT, µMT, and *Cd3e* ^–/–^ mice were euthanized at day 90 p.i., *Rag2* ^–/–^ *Il2rg* ^–/–^ at day 30 p.i., then CFUs in different organs were quantified. (B) Body weight in *C. albicans*‐infected WT (*n* = 5), *Rag2* ^–/–^ *Il2rg* ^–/–^ (*n* = 5), µMT (*n* = 5), and *Cd3e* ^–/–^ (*n* = 5) mice. Shown is the mean ± SD. Statistical significance by the two‐way ANOVA test. (C) Survival curves of *C. albicans*‐infected mice. Statistical significance by the log‐rank test comparison between curves. (D) CFUs in vaginal tissue of *C. albicans*‐infected mice. Shown is the mean ± SD. Statistical significance determined by two‐way ANOVA. (E) CFUs per analyzed organ in *C. albicans*‐infected mice at the time of sacrifice. Shown is the mean ± SD. Statistical significance by one‐way ANOVA test. *p < 0.05, **p < 0.01, ***p < 0.001, ****p < 0.0001. All data are representative of more than three independent experiments with at least three mice per group.

To gain mechanistic insights into the relevance of our findings in humans, we utilized in vivo models of *C. albicans* mucosal infection. First, we defined the role of distinct immune cells in mediating protection in a model of *C. albicans* infection of the vaginal mucosa (Figure 3A). Wild‐type (WT) immunocompetent mice, *Rag2^–/–^Il2rg^–/–^* mice (lacking B and T lymphocytes, innate lymphoid cells [ILCs], and natural killer cells) [27], *Cd3e^–/–^* mice (lacking T cells) [28], and µMT mice (lacking B cells) [29] were administered subcutaneous estrogen on days −7 and 0 to mimic conditions favorable for fungal growth [30]. On days 0 and 1, the animals were intravaginally inoculated with 10^6^ *C. albicans* (laboratory strain 3153a) and monitored over 4 months. Weight loss served as a marker of morbidity, and upon reaching humane endpoints, fungal colony‐forming units (CFUs) were quantified from various organs (Figure 3B–E). WT and µMT mice maintained their weight and rapidly cleared *C. albicans* from the vagina, indicating that B cells are not required for fungal control in this model. *Rag2^–/–^Il2rg^–/–^* mice, which lack ILCs in addition to T and B cells, rapidly lost weight and succumbed to infection within 30 days, displaying a high fungal burden in all tested organs. In contrast, *Cd3e^–/–^* mice initially controlled the infection but began to lose weight around two months post‐infection and succumbed shortly thereafter. These mice exhibited fungal persistence in the vagina for up to 90 days, with dissemination to the stomach and gut (Figure 3D,E). Dissemination likely occurred through coprophagy or grooming, as evidenced by fungal recovery from the intestines of uninfected mice co‐housed with infected animals (Figure S3). These results in a mouse model of vaginal infection demonstrate that while ILCs, either alone or in conjunction with T cells, are critical for early protection against mucosal *C. albicans* infection, T cells are indispensable for long‐term fungal control.

**FIGURE 4:**
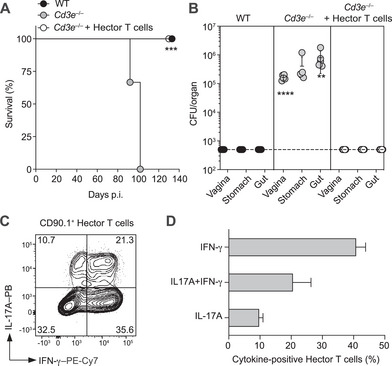
Reconstitution of *Cd3e* ^–/– ^with naïve Hector CD4^+^ T cells restores protection against VVC. CD45.2^+^ *Cd3e* ^–/–^mice were adoptively transferred with 5 × 10^6^ Hector naïve CD4^+^ T cells carrying a different congenic marker (CD45.1 or CD90.1). The day after, WT mice, *Cd3e* ^–/–^ mice, and Hector‐reconstituted *Cd3e* ^–/–^ mice (*n* = 5 mice per group) were infected with *C. albicans*. (A) Survival curves of infected mice. Statistical significance was determined by the log‐rank test comparison between curves. (B) CFUs measured in the vagina, stomach, and gut on day 30 p.i. Shown is the mean ± SD. Statistical significance was determined by one‐way ANOVA test. (C, D) Cytokine production by adoptively transferred CD90.1^+^ Hector CD4^+^ T cells. Cells were recovered from lumbar dLNs on day 12 p.i. and restimulated in vitro for 5 h with PMA and ionomycin in the presence of BFA for the last 2 h. Representative dot plot (C) and mean + SD. of *n* = 3 mice (D). PB = Pacific Blue. Statistical significance determined by one‐way ANOVA test. **p < 0.01, ***p < 0.001, ****p < 0.0001. All data are representative of three independent experiments with 3 to 5 mice per group.

To assess whether CD4^+^ T cells alone are sufficient for protection against *C. albicans*, we adoptively transferred naive T cells from Hector mice, which express a transgenic Vα2/Vβ4 TCR specific to the C2 peptide (residues 126–140) of *C. albicans* alcohol dehydrogenase (ADH1) [23], into *Cd3e^–/–^* mice one day prior to infection. Remarkably, T cell transfer fully restored protection against vaginal *C. albicans* infection, as evidenced by survival and prevention of fungal dissemination to the gastrointestinal tract (Figure 4A,B). Phenotypic analysis of adoptively transferred Hector CD4^+^ T cells from draining lymph nodes (dLNs) at day 12 post‐infection revealed that up to 60% of these cells had differentiated into Th1/Th17 cells (Figure 4C,D).

These experiments confirm that in the mouse model used CD4^+^ T cells are sufficient for protection against mucosal *C. albicans* infection and suggest that IFN‐γ and/or IL‐17 production plays a critical role in fungal control.

### 2.4 Long‐Term Protection Against Mucosal Candidiasis Requires Antigen‐Specific Th17 Cells

Having established that T cells are critical for long‐term protection in our mouse model of mucosal candidiasis, we next investigated the relative contributions of distinct T helper subsets. To this end, Hector CD4^+^ naive T cells were activated in vitro under Th1, Th2, or Th17 polarizing conditions (Figure S4). The resulting effector Th cells were adoptively transferred into *Cd3e^–/–^* mice one day before infection with *C. albicans*.

**FIGURE 5:**
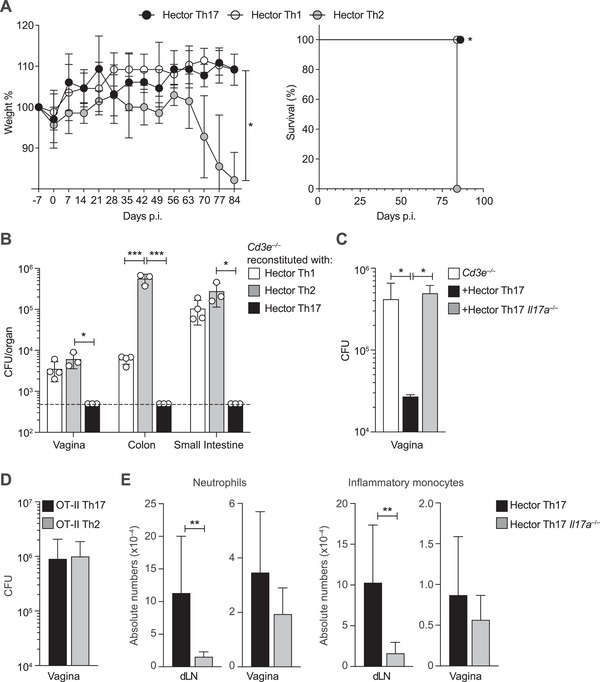
Long‐term protection against *C. albicans* vaginal infection requires IL‐17A^+^ Th17 cells. *Cd3e* ^–/–^ mice were adoptively transferred with 5 × 10^5^ in vitro polarized Hector Th1 (*n* = 4), Th2 (*n* = 3), or Th17 (*n* = 3) cells carrying a different congenic marker 1 day prior to *C. albicans* vaginal infection. (A) Weight loss (left) and survival (right) were monitored over time. Values represent mean ± SD. Statistical significance was determined by two‐way ANOVA test (left) or by a log‐rank test comparison between curves (right). (B) CFUs per gram of tissue (mean ± SD) at the time of sacrifice (day 84 p.i.). The dotted line represents the lower detection limit of CFU in the indicated organs. Statistical significance was determined by one‐way ANOVA test. (C) *Cd3e* ^–/–^ were adoptively transferred with 5 × 10^5^ in vitro polarized Hector Th17 cells or Hector *Il17a* ^–/–^ Th17 cells. CFUs per gram of vaginal tissue (mean + SD) on day 30 p.i. *n* = 3 for each experimental group. *Cd3e* ^–/–^ mice with no T cell transfer served as control. Statistical significance by one‐way ANOVA test. (D) *Cd3e* ^–/–^ mice were adoptively transferred with 5 × 10^5^ in vitro polarized OVA‐specific OT‐II Th2 (*n* = 3) or Th17 (*n* = 3) cells carrying a different congenic marker 1 day prior *C. albicans* vaginal infection. CFU counts in the vaginal tissue were measured on day 30 p.i. Bars represent mean + SD. (E) Absolute number of neutrophils (CD11b^+^, Ly6c^int^, Gr1^+^) and inflammatory monocytes (CD11b^+^, Ly6c^+^, Gr1^int^) in day 30 dLNs (*n* = 10) and vagina (*n* = 6) of *Cd3e* ^–/–^ adoptively transferred with Hector Th17 cells or Hector *Il17a* ^–/–^ Th17 cells. Bars represent the mean + SD. Statistical significance determined by Student's *t*‐test. *p < 0.05, **p < 0.01, ***p < 0.001, ****p < 0.0001. *p < 0.05, **p < 0.01, ***p < 0.001, ****p < 0.0001. All data are representative of three independent experiments with at least three mice per experimental group.

Hector Th17 cells were fully capable of controlling the infection, whereas equal numbers of transferred Hector Th2 cells failed to protect the animals and appeared to exacerbate disease progression, as evidenced by significant weight loss and reduced survival (Figure 5A,B). Although not as effective as Th17 cells, *C. albicans*‐specific Hector Th1 cells partially limited fungal outgrowth and dissemination to the gastrointestinal tract (Figure 5A,B).

The protective effect of Th17 cells against *C. albicans* infection was antigen‐specific and dependent on IL‐17A. Indeed, protection was abolished in mice adoptively transferred with Hector IL‐17A‐deficient Th17 cells (Hector *Il17a^–/–^*) or Th17 cells specific for an irrelevant antigen (OVA‐specific OT‐II) (Figure 5C,D). In the absence of T cell‐derived IL‐17A, the recruitment of Ly6C^+^ Gr1^int^ inflammatory monocytes and Ly6C^int^ Gr1^+^ neutrophils—both of which are considered effector cells in the control of fungal invasion [31]—was reduced in the dLNs and vaginal tissue (Figure 5E). This suggests that Th17 cytokines are essential for orchestrating local fungal control.

Collectively, these findings demonstrate in a model of mucosal infection that antigen‐specific, IL‐17A‐producing Th17 cells are both necessary and sufficient for long‐term protection against *C. albicans*. Furthermore, while Th1 cells offer limited protection, Th2 cells are detrimental, leading to exacerbated disease.

### 2.5 Th2 Cells Impair Th17‐Mediated Protection Against *C. albicans* Mucosal Infection

The observation that Th2‐reconstituted mice exhibited worsened disease, combined with the elevated Th1/Th17 and Th2/Th17 ratios observed in STAT1‐GOF patients, led us to hypothesize that non‐Th17 cells might inhibit or counteract the protective function of Th17 cells. The issue is particularly relevant since the patients, while exhibiting reduced numbers of IL‐17‐producing Th17 cells, still retain some Th17 functionality.

**FIGURE 6:**
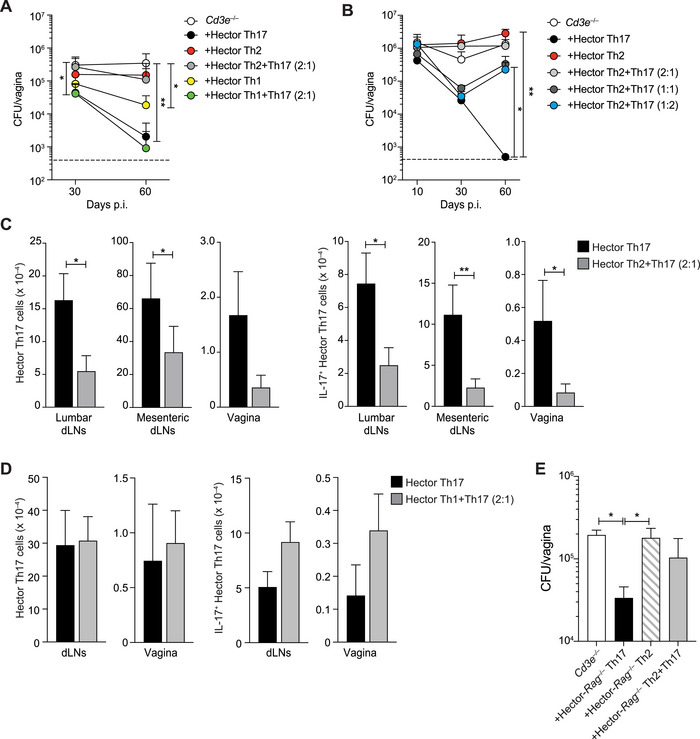
Th2, but not Th1 cells, impair Th17‐mediated protection against *C. albicans* vaginal infection. *Cd3e* ^–/–^ mice were reconstituted one day prior to vaginal *C. albicans* infection with Hector Th17, Th1, or Th2 cells carrying different congenic markers or with combinations of Hector Th1 and Hector Th17 or Hector Th2 and Hector Th17 (2 to 1 ratio) cells. *Cd3e* ^–/–^ mice with no T cell transfer served as control. (A) CFUs (mean + SD) per gram of vaginal tissue measured on days 30 and 60 p.i. (*n* = 6 for each time point and group). Statistical significance determined by a one‐way ANOVA test. (B) *Cd3e* ^–/–^ mice were reconstituted with Hector Th17 or Hector Th2 cells or with combinations of Hector Th2 and Hector Th17 cells at different ratios. CFUs (mean + SD) per gram of vaginal tissue on day 10, 30, and 60 p.i. (*n* = 3 for each time point and group). Dotted line in (A) and (B) represents the lower detection limit of *C. albicans* CFU. Statistical significance determined by one‐way ANOVA test. (C) Absolute number (mean + SD) of Hector Th17 cells (left) and Hector IL‐17A^+^ Th17 cells (right) recovered from the lumbar (*n* = 3) and mesenteric (*n* = 4) dLNs and from the vagina (*n* = 3) of *Cd3e* ^–/–^ mice reconstituted with Hector Th2 and Hector Th17 cells (2 to 1 ratio) on day 30 p.i. Statistical significance determined by Student's *t*‐test. (D) Absolute number (mean + SEM) of Hector Th17 cells (left) and Hector IL‐17A^+^ Th17 cells (right) recovered from dLNs (*n* = 12) and vagina (*n* = 6) of *Cd3e* ^–/–^ mice reconstituted with Hector Th1 and Hector Th17 cells (2 to 1 ratio) on day 30 p.i. To determine production of IL‐17A, T cells were restimulated in vitro for 5 h with PMA and ionomycin in the presence of BFA for the last 2 h followed by intracellular cytokine staining. Statistical significance determined by Student's *t‐*test. (E) CFUs (mean + SD) on day 30 p.i. in the vaginal tissue of *C. albicans*‐infected *Cd3e* ^–/–^ mice, which were reconstituted with in vitro polarized Hector Th cells obtained from transgenic mice on *Rag1* ^–/–^ background (*n* = 3 per group). Statistical significance determined by one‐way ANOVA test. *p < 0.05, **p < 0.01, ***p < 0.001, ****p < 0.0001. All data are representative of at least three independent experiments (2 in D) with a minimum of three mice per experimental group.

To address this issue, we reconstituted *Cd3e^–/–^* mice with Hector Th1, Th2, or Th17 cells, either individually or in combinations, and subsequently infected the mice with *C. albicans*. Control groups included *Cd3e^–/–^* mice infected without reconstitution. Consistent with prior findings, Th17 cells alone achieved efficient control of *C. albicans* colonization in the vagina by day 60. In contrast, Th2 cells alone did not, and Th1 cells provided only partial control of fungal growth (Figure 6A). Notably, the presence of Th1 cells did not interfere with Th17‐mediated protection, while the addition of Th2 cells completely abolished this protection (Figure 6A). The detrimental effect of Th2 cells was proportional to their initial abundance relative to Th17 cells and was evident even at a reduced Th2‐to‐Th17 cell ratio (1:2) (Figure 6B).

Analysis of cell counts in the draining lymph nodes (mesenteric and lumbar) and vaginal tissue revealed a significant reduction in the number of Hector Th17 cells and IL‐17A^+^ Th17 cells in mice co‐transferred with Hector Th2 cells, whereas no such reduction occurred in mice co‐transferred with Hector Th1 cells (Figure 6C,D). The negative impact of Th2 cells on Th17 cells was also reflected in the abolishment of fungal control by Th17 cells when co‐transferred with Th2 cells (Figure 6E). Despite the well‐documented plasticity of T cells, Hector Th17 cells recovered from the dLNs and vaginal tissue retained their IL‐17A production capacity at 10‐ and 30 days postinfection, regardless of the presence of Th2 cells (Figure S5A,B)

To assess whether the suppressive effect of Th2 cells on Th17 cells extends to other mucosal tissues commonly affected in CMC, we used a model of oropharyngeal candidiasis (OPC). *Cd3e^–/–^* mice were reconstituted with different combinations of Hector Th17 and Hector Th2 cells prior to oral *C. albicans* colonization. Fungal burden in the tongue was measured over time. Consistent with results from the vaginal candidiasis model, only mice reconstituted with Hector Th17 cells alone successfully controlled the fungus (Figure S6A). Mice receiving Hector Th2 cells alone or in combination with Th17 cells failed to control the infection. Furthermore, Hector Th2 cells impaired the ability of Hector Th17 cells to produce IL‐17, exacerbating the failure to control *C. albicans* in the oral cavity (Figure S6B–D).

These findings demonstrate that Th2 cells impair the control of *C. albicans* infection at mucosal surfaces by restricting Th17 cell expansion and, in some tissues, suppressing their effector functions, ultimately compromising protective immunity.

### 2.6 Th2 Cells Suppress Th17 Protective Function Through IL‐4 Secretion

**FIGURE 7:**
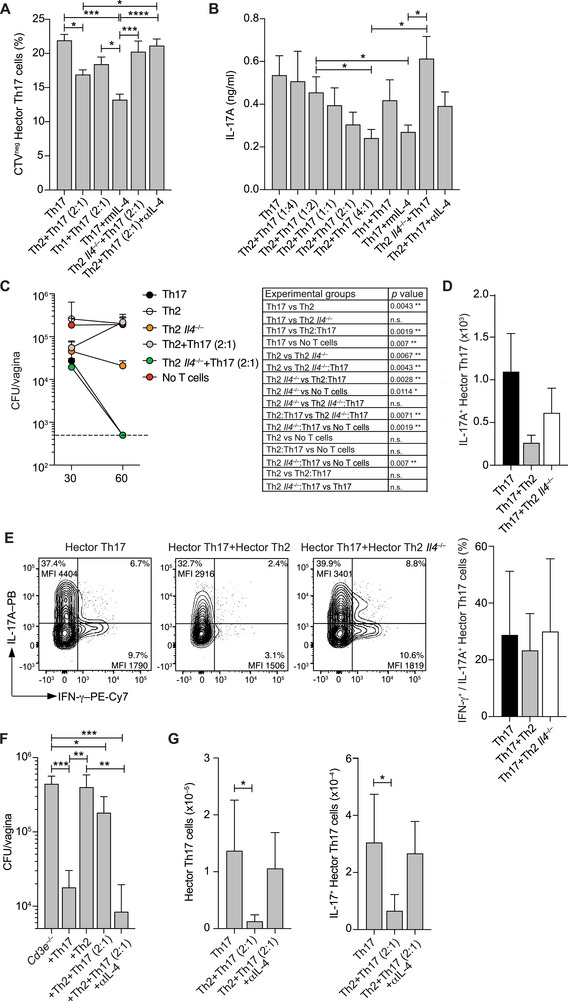
IL‐4 neutralization rescues the capability of Hector Th17 cells to protect against *C. albicans* vaginal infection. (A, B) *In vitro* proliferation assay of and cytokine production by CTV‐stained Hector Th17 cells cultured alone or co‐cultured with Hector Th2, Hector Th1, or Hector *IL4^–/–^* Th2 cells. Th17 cells were also cultured with the addition of rmIL‐4 to the cell medium or together with Th2 cells plus an αIL‐4 monoclonal antibody (mAb). Proliferation was measured as the dilution of CTV after 72 h of culture. Cytokines were measured in the 72h‐supernatants by ELISA. Bars represent mean + SEM and are the pool of *n* = 3 independent experiments. Statistical significance was determined by a one‐way ANOVA test. (C) CFUs in the vagina of *Cd3e* ^–/–^ mice reconstituted with Hector Th17, Hector Th2, or Hector *IL4^–/–^* Th2 cells or with combinations of Hector Th2 and Hector Th17 (ratio of 2 to 1) or of Hector *IL4^–/–^* Th2 and Hector Th17 (2 to 1). Vaginal tissue was analyzed on days 30 and 60 p.i. *n* = 5 for each experimental group. Values represent the mean + SD, dotted lines represent the lower detection limit of CFU/gram of organ. Statistical significance determined by the one‐way ANOVA test is shown in the right panel. (D) Absolute numbers on day 30 p.i. of Hector IL‐17A^+^ Th17 cells recovered from the vagina of *Cd3e* ^–/–^ mice reconstituted with Hector Th17 cells or combinations of Hector Th2 and Hector Th17 (ratio of 2 to 1) or Hector *IL‐4^–/–^ Th2* cells and Hector Th17 (ratio of 2 to 1) (*n* = 10 for each experimental group). Bars represent mean + SD. (E) Representative FACS plot of intracellular cytokine staining of Hector Th17 cells recovered from the vagina of infected mice in (D) on day 30 p.i. For intracellular cytokine staining, Th17 cells were restimulated in vitro for 5 h with PMA and ionomycin in the presence of BFA for the last 2 h. The histogram on the right shows percentages of IFN‐γ^+^ and/or IL‐17A^+^ Th17 cells in the different experimental settings; values and are the mean + SD of three independent experiments. MFI = mean fluorescence intensity, PB = Pacific Blue. (F, G) *Cd3e* ^–/–^ were reconstituted with in vitro polarized Hector Th17, Hector Th2, or a combination of Hector Th2 and Hector Th17 (ratio of 2 to 1) 1 day before vaginal *C. albicans* infection. *Cd3e* ^–/–^ with no T cell transfer served as control. A group of mice receiving Th2 and Th17 cells was treated with αIL‐4 neutralizing mAb or with an isotype control mAb every second day until the end of the experiment (day 30 p.i.). Shown are the CFUs per gram of vaginal tissue (F) and the total number of Hector Th17 cells (left) and of Hector IL‐17A^+^ Th17 cells (right) in lumbar dLNs (G) in the different experimental groups (*n* = 4–5 for each group) on day 30 p.i. Bars represent the mean + SD. Statistical significance determined by one‐way ANOVA test. *p < 0.05, **p < 0.01, ***p < 0.001, ****p < 0.0001. All data are representative of at least two independent experiments.

Our findings that enhanced Th2 responses impair Th17‐mediated protection led us to hypothesize that Th2 cells exert their detrimental effects through cytokine secretion. Several lines of evidence support a role for IL‐4 and other Th2 cytokines in suppressing Th17 cell differentiation and function [32, 33, 34, 35]. By inducing STAT6, IL‐4 suppresses IL‐23 transcription and secretion by antigen‐presenting cells [36] and interferes with STAT3 binding to the *Il17a* promoter, thereby disrupting the Th17 transcriptional program, particularly at early stages of differentiation [33]. Supporting our hypothesis, an in vitro co‐culture system revealed that Hector Th2 cells significantly reduced both Hector Th17 cell proliferation and IL‐17A production in a dose‐dependent manner (Figure 7A,B). In contrast, neither Hector Th1 cells nor Hector IL‐4‐deficient Th2 cells affected Th17 activation and function. Adding recombinant IL‐4 to Th17 cultures similarly decreased proliferation and IL‐17A production, whereas neutralizing IL‐4 in a Th12‐Th17 co‐culture restored Th17 proliferation and, partially, IL‐17A production (Figure 7A,B). These in vitro results support the notion that IL‐4 inhibits Th17 cells.

To validate this mechanism *in vivo*, we transferred Hector Th17 cells, Hector Th2 cells, or Hector IL‐4‐deficient Th2 cells into *Cd3e^–/–^* mice, either alone or in combination, prior to *C. albicans* infection. Remarkably, mice receiving Hector Th17 cells alone or in combination with IL‐4‐deficient Th2 cells cleared *C. albicans* from the vagina by day 60 postinfection (Figure 7C). In these mice, Hector IL‐17A^+^ Th17 cells expanded robustly in vaginal tissue and maintained IL‐17 production capacity (Figure 7D,E). Similarly, in *C. albicans*‐infected *Cd3e^–/–^* mice co‐transferred with Hector Th2 and Th17 cells, treatment with an IL‐4‐neutralizing antibody (αIL‐4) significantly improved fungal control, reducing CFU counts in vaginal tissue to levels comparable to those in mice reconstituted with Th17 cells alone (Figure 7F). Neutralization of IL‐4 also restored expansion of and IL‐17A production by Hector Th17 cells (Figure 7G).

Collectively, these findings demonstrate that, during *C. albicans* infection, IL‐4 derived from Th2 cells directly inhibits Th17 cells, suppressing their proliferation and IL‐17A production, thereby impairing fungal control. This pathological effect can be reversed by blocking IL‐4, highlighting a potential therapeutic avenue for restoring protective immunity in diseases characterized by Th2‐mediated suppression of Th17 function.

## 3 Discussion

Failure to mount an appropriate T cell response, resulting in the generation of T helper cell subsets that are not optimally suited for a specific pathogen, can lead to ineffective immunity and pathological outcomes. A well‐known example, is the Th1/Th2 paradigm observed in leprosy, where patients capable of mounting a strong Th1 response, can effectively limit bacterial replication, generating a less severe form of disease (Tuberculoid leprosy) while those in which responses are dominated by Th2 cells, which suppress effective Th1‐mediated defense, allow for bacterial persistence and widespread infection, producing a deadlier form of the disease (lepromatous leprosy) [37]. Our findings demonstrate that while *C. albicans*‐specific Th17 cells are essential for fungal control, alternative Th responses, particularly Th1 and Th2, are ineffective and, in the case of Th2, may contribute to disease susceptibility in both humans and mice.

Patients with inborn errors in key immune pathways often display increased susceptibility to specific pathogens [17]. In particular, patients with CMC are generally characterized by impaired IL‐17A/IL‐17F immunity, consistent with the notion that the IL‐17 pathway is essential for protective immunity against fungi [9]. In all known cases of CMC associated with impaired IL‐17 immunity—whether due to defective IL‐17 production or signaling (e.g., mutations in *IL17RA* or *IL17RC*)—multiple immune cell types are affected. Although no primary immunodeficiency has been identified that exclusively disrupts Th17 function, our findings suggest that Th17 cells play a particularly critical role in antifungal immunity, potentially even acting as an essential and nonredundant subset in mucosal *C. albicans* defense. This raises the possibility that a yet‐to‐be‐discovered genetic defect could specifically impair Th17 cells and underlie CMC in a subset of patients. While our study focused on the adaptive T cell response induced by *C. albicans* in STAT1‐GOF patients, we cannot exclude differences in the innate immune recognition of specific fungal cell wall components, such as glucan, mannan, and chitin, that may influence the cytokine milieu and thus impact Th cell polarization.

Using blood samples from CMC STAT1‐GOF patients and mouse models of mucosal infection, we investigated whether *C. albicans*‐specific Th cell subsets not typically associated with fungal sterilizing immunity (i.e., Th1 and Th2) are generated, recognize similar antigens, and contribute to either protection or exacerbation of disease, alone or in combination with protective Th17 cells. Our findings expand on existing knowledge by providing evidence that in both human and animal models, a qualitative alteration in the Th cell profile, without measurable changes in response magnitude or antigenic specificity, compromises immunity to *C. albicans* infection. Specifically, Th2 responses may lead to detrimental outcomes and contribute to chronic disease progression.

Patients’ T cells appear to recognize antigens similar to those recognized by healthy individuals. To investigate this in detail, we generated what we believe to be the first comprehensive antigenic fingerprint of *C. albicans*‐specific T cell responses, screening an extensive set of peptide pools representing 80 proteins known to be abundant and either immunogenic or relevant to pathogenicity or fungal biology. This assay enabled a direct comparison of the relative immunodominance of both well‐characterized and previously unrecognized *C. albicans* antigens. These include HYR1 and SAP4‐6, which show frequencies of recognition by T cells equal to or greater than the reference immunodominant antigen MP65.

Responses to the top three *C. albicans* antigens, as well as to a peptide pool representing the 15 most immunodominant *C. albicans* antigens, were quantitatively similar in patients and controls. However, in patients with STAT1‐GOF mutations, the Th profile showed a completely inverted pattern of dominance: responses were primarily driven by Th1 and Th2 cells, whereas in healthy individuals, Th1* and Th17 cells predominated. This is consistent with previous reports indicating that, in healthy individuals, Th cells producing protective IL‐17 and capable of mucosal migration via CCR6 dominate *C. albicans‐*specific responses [24].

While T cell responses in STAT1‐GOF patients are typically associated with enhanced type I immunity, our sensitive peptide‐based T cell assay revealed a significant presence of *C. albicans*‐specific Th2 cells. Although the absolute numbers of these Th2 cells may not exceed those observed in healthy individuals, our findings suggest that the relative ratio between Th2 and Th17 cells could ultimately influence infection outcomes. Specifically, the predominance of Th2 cells might have a disproportionately adverse impact on individuals with reduced Th17 responses, thereby tipping the immune balance away from effective fungal control. The elevated frequency of *C. albicans*‐specific Th1 cells observed in STAT1‐GOF patients may serve as a compensatory mechanism for impaired Th17 responses, potentially mitigating some of the adverse effects associated with an elevated frequency of *C. albicans*‐specific Th2 cells. Future investigations should examine whether similar imbalances in the Th cell repertoire occur at mucosal sites in these patients, as well as whether the antigenic profiles and frequencies observed in circulation are preserved in the tissues.

To gain mechanistic insights into findings from patients with STAT1‐GOF mutations, we used different mouse models of mucosal candidiasis. Although *C. albicans* is not a mouse commensal, this approach allowed us to investigate the impact of different immune cell populations, including T cells with distinct phenotypes. Our findings indicate that, among various genetic backgrounds tested, *Rag2* ^–/–^ *Il2rg* ^–/–^ mice and *Cd3e* ^–/–^ mice inoculated with *C. albicans* fail to control the infection, highlighting the essential role of both ILCs and T cells in containing fungal spread [15, 38]. In particular, *Rag2* ^–/–^ *Il2rg* ^–/–^ mice experienced a severe and rapid disease progression with high pathogen burdens, underscoring the critical role of ILCs as a first line of defense against *C. albicans*. Notably, *Cd3e* ^–/–^ mice were also unable to control *C. albicans* dissemination and succumbed at a later time point, emphasizing the importance of T cell immunity for pathogen control at mucosal surfaces. Th17 cells were essential for protection, and antigen‐specificity was required, aligning with a classical T cell activation model in this context. In agreement with previous literature [14, 39], our data suggest that type 3 immunity is crucial in recruiting inflammatory myeloid cells, such as neutrophils and monocytes, which are established key players in the clearance of *C. albicans* and other fungi [39, 40, 41, 42, 43].

In the model used, Th1 and Th2 cells appeared either nonprotective or detrimental. IFN‐γ production did not seem to negatively impact the overall response and contributed to some level of fungal containment with several lines of evidence supporting this notion. First, naïve Hector CD4^+^ T cells, when transferred into *C. albicans*‐infected *Cd3e* ^–/–^ mice, which provided protection, acquired an IFN‐γ^+^/IL‐17^+^ phenotype. Second, in vitro‐polarized IFN‐γ^+^ Hector Th1 cells, when transferred into *C. albicans*‐infected *Cd3*e^–/–^ animals, achieved only a partial fungal containment, but prevented the development of overt disease. This suggests that type 1 immunity may potentially promote a state of tolerance, which can be considered as a favorable outcome if sterilizing immunity cannot be achieved [44]. Additionally, upon co‐transfer, Hector Th1 cells showed poor expansion and did not affect the proliferation or functions of Hector IL‐17A^+^ Th17 cells. While none of the known inborn errors of immunity associated with impaired or abolished responses to IFN‐γ lead to *C. albicans* infections [45], antigen‐specific impairment of IFN‐γ production has been reported in women with recurrent vaginal candidiasis [46, 47]. It is worth noting that recent research highlighted a cytotoxic and potentially pathogenic role for fungus‐specific Th1 cells in inflammatory bowel disease (IBD) patients [48]; further studies are needed to assess the properties of Th1 cells generated in this context and their specific impact at mucosal surfaces.

In contrast to Th1 cells, Th2 cells appeared detrimental, worsening host conditions whether administered alone or together with protective Th17 cells. When transferred alone, Hector Th2 cells lead to higher fungal burdens and increased pathology. To more accurately replicate the Th cell distribution observed in patients, which retain a reduced yet present Th17 compartment, we co‐transferred *C. albicans*‐specific Th2 and Th17 cells into *Cd3e* ^–/–^ mice at different ratios. Strikingly, we found that even at a 1:2 ratio, Th2 cells could abolish the protective effects of Th17 cells, suggesting that the relative ratio within the Th compartment may be more crucial than absolute numbers in determining response outcomes. In two distinct models of mucosal infection, namely vaginal and oropharyngeal, we observed that Th2 cells could limit Th17 expansion/survival, IL‐17 production, or both. Mechanistically, we demonstrated both in vitro and in vivo that Th2 cells exert a negative impact on Th17 proliferation and function via secretion of IL‐4. Th2‐related cytokines are known to negatively regulate IL‐17A expression and Th17 cell expansion by affecting RORγt expression and increasing STAT6 and GATA3 phosphorylation [32]. IL‐4 downregulates IL‐17 by inhibiting STAT3 binding to the *Il17a* promoter [33]. Crucially, IL‐4 neutralization restored normal expansion and IL‐17A production in *C. albicans*‐specific Th17 cells in a Th2–Th17 co‐transfer model in vivo, resulting in protective immunity, indicating a potentially effective approach to mitigate pathological outcomes associated with altered antigen‐specific Th cell distributions in patients. Additional research is required to determine whether anti‐IL‐4 therapy could be applied in STAT1‐GOF patients to promote proper Th17 cell expansion and enhance control of chronic *C. albicans* infections. It is worth noting that Th2 cells are not the only source of type 2 cytokines in vivo, as ILCs are also significant producers [49].

The regulatory effects of type 2 cytokines may extend beyond those observed in this study on Th17 cells, potentially impacting epithelial and stromal cells as well. Epithelial cells express STAT3, which regulates several antimicrobial peptides and protective factors [50], becoming then an important target of Th2‐cytokines during the first reaction to *C. albicans* infection [51, 52]. Additionally, the presence of Th2 cells could influence the expansion and cytokine production of type 3 ILCs responding to infection in the T‐cell‐deficient immunocompromised host [15]. This potentially points to a broader role for type 2 cytokines in these settings.

Overall, our study suggests that detrimental effects may be exerted by Th2 cells in CMC patients. The altered distribution of fungus‐specific Th cells, dictated by genetic alteration, in response to *C. albicans* colonization, is unable to correctly respond to the infection, and it is also affecting the protective role of IL‐17A^+^ Th17 cell subset. We propose that IL‐4 neutralization may represent a putative strategy to improve disease outcome in chronically affected patients characterized by an altered distribution of the *C. albicans*‐specific Th‐cell subsets.

## 4 Material and Methods

### 4.1 Human Subjects

Patients and their families included in the study are described in Table S1 and in other studies [22, 53]. The studies were approved by the institutional review boards of the centers at which the patients were managed. Informed consent was obtained from the patients or their parents (for minors). Blood from healthy individuals was obtained from the Swiss Blood Donation Center of Basel and Lugano and used in compliance with the Federal Office of Public Health (authorization no. A000197/2 to F.S).

### 4.2 Human T Cell Subsets and Cloning

Peripheral blood mononuclear cells (PBMCs) were obtained by gradient separation using Ficoll‐Paque Plus (GE Healthcare). CD14^+^ monocytes and total CD4^+^ T cells were isolated by positive selection using magnetic microbeads. Memory T helper cell subsets were then identified and sorted to over 97% purity according to the differential expression of CXCR3, CCR4, and CCR6, and after gating on CD8^–^CD14^–^CD16^–^CD19^–^CD25^–^CD56^–^CD45RA^–^ cells, as previously described [24]. The following monoclonal antibodies (mAbs) were used for staining: anti‐CD45RA‐FITC (ALB11), anti‐CD45RO‐PE (UCHL1), anti‐CD8‐PE‐Cy5 (B9.11), anti‐CD14‐PE‐Cy5 (RMO52), anti‐CD16‐PE‐Cy5 (3G8), anti‐CD19‐PE‐Cy5 (J3‐119), anti‐CD25‐PE‐Cy5 (B1.49.9), anti‐CD56‐PE‐Cy5 (N901) (all from Beckman Coulter), anti‐CCR6‐PE (11A9), anti‐CCR4‐PE‐Cy7 (1G1) (BD Biosciences), anti‐CXCR3‐AlexaFluor 647 (G025H7), anti‐CCR7‐BV421 (G043H7), anti‐CD95‐APC (DX2) (Biolegend), anti‐CD19‐FITC (HIB19) (BD Biosciences), anti‐CD45RA‐BV650 (MEM‐56), anti‐CD4‐PE‐CF594 (S3.5) (Thermo Scientific). Cells were stained on ice for 15–20 min and analyzed and sorted with FACSAria III (BD Biosciences). To obtain T cell clones, sorted T cells were suspended in RPMI 1640 medium supplemented with 2 mM glutamine, 1% nonessential amino acids, 1% sodium pyruvate, 1% penicillin/streptomycin (all from Life Technologies). Cells were plated at 0.5 cells per well in 384‐well plates in the presence of irradiated allogenous PBMCs (2.5 × 10^4^ cells per well), IL‐2 (500 units/mL), and phytohemagglutinin (PHA, Remel) (1 µg/mL). Cells were expanded for 12 days, and wells with dividing cells were further expanded for downstream analyses.

### 4.3 T Cell Library Assay

The assay was performed as previously described [26]. Briefly, T cells (500–2000 cells/well, concentration and number of wells depending on the subset and number of sorted cells) were polyclonally expanded in the presence of 1 µg/mL PHA (Remel, Thermo Scientific), irradiated (45 Gy) allogeneic feeder cells, and IL‐2 (500 IU/mL). After 14–21 days, T cells from each well were extensively washed and stimulated with autologous monocytes pulsed with the antigens of interest. Proliferation was assessed at day 4 after 16 h incubation with 1µCi/mL [3H] Thymidine (GE Healthcare). Precursor frequencies were calculated based on the number of wells that scored negative for proliferation according to the Poisson distribution and expressed per million cells.

### 4.4 Peptide Bioinformatic Analyses and Production

Eighty *C. albicans* proteins with different cellular locations and functions (cytosolic enzymes, cell‐wall‐associated structural and enzymatic proteins, extracellular hydrolases) were selected based on their abundance or known to be either immunogenic or relevant to pathogenicity or fungal biology (Table S2). 15‐mer peptides overlapping by 10, spanning each sequence, were extracted using bioinformatics tools (8059 15‐mers in total). Each peptide was scored for predicted binding to a panel of 24 common HLA class II DR, DP, and DQ molecules for which binding assays are available. For 23 of the molecules, the peptides were scored utilizing the recommended IEDB consensus algorithm; for one molecule (DRB3*02:02), the IEDB‐recommended method was NetMHCIIpan. For each molecule, we utilized a consensus score (percentile rank) ≤20.0 to define a binder. Finally, a set of 1273 peptides was selected corresponding to those peptides with consensus percentile rank ≤20.0 for 12 or more of the 24 class II molecules probed. These selection criteria were similar to those used in other studies [54, 55]. Each protein was represented by a minimum of 4 to a maximum of 51 peptides (average 16.8). The peptides were synthesized by A and A (San Diego, CA) as crude material on a small (1 mg) scale, individually resuspended in DMSO, and pooled accordingly to the belonging protein. Individual peptides and pools were divided into aliquots and stored at −80°C.

### 4.5 Functional Assays of Human T Cells

CD4^+^ CD45RO^+^ memory T cells were obtained from PBMCs by negative selection with bead‐based enrichment, CFSE‐labeled, and co‐cultured with autologous monocytes (2:1 ratio) preincubated with 1 µg/mL peptide pool for 2.5 h in U‐bottom 96‐well plates. After 7 days, 50 U/mL IL‐2 was added to the culture, and CFSE dilution was assessed after an additional 5 days. Each peptide pool was tested in duplicate for each individual; five individuals were tested; a significant response was defined as % CFSE^lo^ > 5× background proliferation in the absence of peptide stimulation.

### 4.6 Mice

*Rag2* ^–/–^ *Il2rg* ^–/–^ [27], *Cd3e* ^–/–^ [28], and Hector [23] mice have been described previously. C57BL/6J (wild‐type, WT) mice were obtained from Charles River Italy Srl. µMT [29] and *Il4* ^–/–^ [56] mice were purchased from Jackson (JAX 002288 and JAX 002518). *Il4* ^–/–^ mice were then crossed in our SPF facility with Hector TCR transgenic mice to obtain *Il4* ^–/–^ Hector CD4^+^ T cells. To identify transferred cells within hosts, donor Hector mice were bred onto a CD45.1 or CD90.1 allele background. All mice were bred and maintained under specific pathogen‐free conditions. Mice were housed in ventilated cages under standardized conditions (20 ± 2°C, 55 ± 8% relative humidity, 12 h light/dark cycle). Food and water were available ad libitum, and mice were examined daily. Female mice were used between 5 and 10 weeks of age. Animals were treated in accordance with the guidelines of the Swiss Federal Veterinary Office. Mouse experiments were performed according to the Swiss Federal Veterinary Office Guidelines and were approved by the Cantonal Veterinary Office (approval no. 28608 TI34/16).

### 4.7 Cell Preparation and in Vitro Polarization for Adoptive Transfer in *Cd3e* ^–/–^ Mice

To isolate naïve T cells from Hector CD90.1^+^, Hector CD45.1^+^, or *Il4* ^–/–^ Hector mice, cells from the spleen and lymph nodes (LNs) were first enriched with anti‐CD4 magnetic beads (L3T4, Miltenyi Biotec) and then sorted on a FACSAria Cell Sorter (BD Biosciences) to obtain cells with a CD4^+^CD8^−^CD25^−^CD44^lo^CD62L^hi^ phenotype. In some experiments, naïve CD4^+^ T cells were then transferred intravenously (i.v.) in estrogen‐treated *Cd3e* ^–/–^ mice the day before *C. albicans* infection. In some experiments, before adoptive transfer, FACS‐sorted naïve T cells were cultured for 2 days in NUNC 96‐well MicroWell MaxiSorp plates (Sigma‐Aldrich M9410) coated with αCD3/αCD28 (2 µg/mL) antibodies and in the presence of cytokine polarizing cocktails. On day 2, cells were transferred to U‐bottom plates and cultured for 2 additional days in the presence of recombinant IL‐2 (500 U/mL). For Th1 polarization, naïve Hector CD4^+^ T cells were cultured with 20 µg/mL of rmIL‐12 (410‐ML R&D Systems), for Th2 polarization with 10 ng/mL of rmIL‐4 (404‐ML R&D System), for Th17 polarization with 25 ng/mL of rmIL‐6 (216‐16 PreproTech), 20 ng/mL of rmIL‐1β (575104 BioLegend), 25 ng/mL of rmIL‐23 (1887‐ML R&D System), and 2 ng/mL of rm‐TGFβ (766 MB R&D System). Polarized Th cells were extensively washed with PBS before transfer into the *Cd3e* ^–/–^ host mice.

### 4.8 VVC Infection Model, CFU Count, and Organ Processing

For *C. albicans* infection, an already established protocol [30] was followed with some modifications to obtain a homogenous group of infected mice in terms of hormone cycles and to render the vaginal tissue more prone to *C. albicans* colonization. Briefly, β‐estradiol 17‐valerate (Sigma E1631, 200 µg/mouse) was solubilized in Ethanol 100% and brought to a final volume of 100 µL/mouse in sesame oil (Supplied by Sigma S3547). Isoflurane‐anesthetized animals were injected subcutaneously (s.c.) in the dorsal back on day −7 before *C. albicans* vaginal inoculation and on day 0 (the same day of the first infection dose). *C. albicans* strain 3153a, which has been reported to be optimal for induction of vaginal infection [30], was cultured in YPD medium for 16 h at 37°C on a rotator. Cultured conidia were confirmed to be in log phase growth by λ660 nm O.D. measurement, then washed twice with PBS, and delivered into the vagina of asleep animals in a volume of 10 µL, 5 × 10^5^ conidia cells per mouse, for two consecutive days (10^6^ conidia cells/mouse in total). Conidia cell number was determined through O.D. measurement, considering that O.D. = 1 corresponds to 10^7^ conidia cells/mL. Mice were weighed weekly and euthanized accordingly to humane endpoint criteria. Organs and tissues were removed from sacrificed animals, homogenized, and seeded in serial dilution on YPD agar plates containing Ampicillin 100 µg/mL (Sigma A9518). CFUs were assessed after 36 h of culture at 37°C and normalized for organ weight. To recover transferred T cells from the vaginas, organs were cut into fine pieces and digested with DNase I 0.1 mg/mL (PanReac AppliChem A3778) and Liberase TM 0.25 mg/mL (Roche 05401127001) in PBS at 37°C for 50 min. Single cell suspensions were passed through a 40 µm strainer using PBS supplemented with 1% FBS and 2 mM EDTA. In some experiments, *C. albicans* infected mice were treated with the αIL‐4 neutralizing antibody (BioXCell, clone 11B11, 0.5 mg i.p./mouse) or with the rat IgG1 isotype control antibody (BioXCell, clone TNP6A7) diluted in the *InVivo*Pure pH7.0 Dilution Buffer (IP0070), two times a week starting from the same day of the T‐cell transfer until the end of the experiment.

### 4.9 OPC Infection

*C. albicans* isolate 101 [57] was cultured in YPD medium for 16 h at 30°C, 180 rpm. Yeast cells were washed twice with PBS. Mice were infected sublingually with 2.5 × 10^6^ yeast cells per mouse without immunosuppression [58]. In brief, mice were anesthetized by injection of 100 mg/kg Ketamine and 20 mg/kg Xylazine in sterile saline i.p., administered in two doses. *C. albicans* was administered by depositing a 2.5 mg cotton ball that was soaked in 100 µL yeast cell suspension under the tongue for 75–90 min. Mice were kept on a heating mat at 35–37°C during the entire period of anesthesia, administered with 10 mL/kg sterile saline to stabilize the circulation, and treated with vitamin A ointment to avoid desiccation of the eyes. Yeast cell number was determined through O.D. (600 nm) measurement, considering that O.D. = 1 corresponds to 10^7^ conidia/mL. Mice were weighed weekly and euthanized accordingly to humane endpoint criteria. Tongues were removed from sacrificed animals, homogenized in sterile water supplemented with 0.05% NP40 in H_2_O, and a 5 mm steel ball for 3 min at 25 Hz using a Tissue Lyzer (Qiagen). Serial dilutions were plated on YPD agar. CFUs were assessed after 36 h of incubation at 30°C.

### 4.10 In Vitro Assay

In vitro polarized Hector Th cells were co‐cultured with irradiated splenocytes from *Rag1* ^–/–^ mice pulsed with C2, pADH1126‐140 (100 ng/mL) peptide plus 5 × 10^3^ particles/mL of heat‐killed *C. albicans*. Cell Trace Violet (CTV, Invitrogen C34557) labelled 10^4^ Hector Th17 cells were co‐cultured for 72 h with twice the number of Hector Th2 or Th1 cells, in the presence of rmIL‐4 (10 µg/mL), or with Th2 cells + αIL‐4 neutralizing antibody (10 µg/mL).

For the ELISA assay, CTV‐labelled 10^4^ Hector Th17 cells were also co‐cultured for 72 h with Hector Th2 cells in the ratio Th2:Th17 of 1:4, 1:2, 1:1, 2:1, or 4:1.

### 4.11 Flow Cytometry Analysis

For analysis of mouse cell phenotypes, the following mAbs were used: anti‐CD3 (clone: 145‐2C11), anti‐CD4 (RM4‐5), anti‐CD8a (53–6.7), anti‐CD25 (PC61), anti‐CD44 (IM7), anti‐CD62L (MEL14), anti‐CD90.1 (OX‐7), anti‐CD45.1 (A20), anti‐CD45.2 (104), anti‐CD90.2 ((30)‐H12), anti‐CD11c (HL3), anti‐CD11b (M1/70), anti‐Ly6C (HK1.4), anti‐Ly6G (1A8), anti‐F4/80 (BM8), anti‐IFN‐γ (XMG1.2), anti‐IL‐17A (TC11‐18H10), anti‐IL‐4 (11B11), anti‐IL‐13 (eBio13A), anti‐IL‐5 (TRFK5). Dead cells were always excluded by staining with 7‐AAD (BioLegend) or 4,6‐diamidino‐2‐phenylindole (DAPI, Sigma). All antibodies were used at 1 µg per 10^6^ cells. For intracellular cytokine staining, cells were stimulated for 5 h with phorbol 12‐myristate 13‐acetate (PMA, 10^−7^ M; Sigma) and ionomycin (1 µg/mL; Sigma), in the presence of brefeldin A (BFA, 10 µg/mL; Sigma) for the last 2 h of culture. Cells were fixed with 4% (wt/vol) paraformaldehyde and permeabilized with 0.5% (wt/vol) saponin (Sigma). Eight‐color staining was performed with the appropriate combinations of antibodies conjugated to fluorochromes. Samples were acquired on a FACSCanto II (BD Biosciences) and analyzed with FlowJo software (TreeStar).

### 4.12 ELISA Assay

To measure cytokine concentrations in cell culture supernatants, the Ready‐Set‐Go! (eBioscience) ELISA kits used: mouse IFN‐γ, IL‐17A, IL‐13, and IL‐5. ELISA assays were performed according to the manufacturer's instructions.

### 4.13 Statistics

Data were analyzed with Prism 5 (GraphPad Software) using the Log‐rank test, comparison between curves, Student's *t*‐test, two‐way ANOVA, and one‐way ANOVA statistical analysis. Graphs show the mean + standard deviation (SD) or standard error of the mean (SEM). *p < 0.05, **p < 0.01, ***p < 0.001, ****p < 0.0001.

## Author Contributions

Camilla Basso, Corinne De Gregorio, Roberta Marzi, Florian Kirchner, Gabor Gyülveszi, Simone Becattini, and Mélanie Migaud performed experiments, acquired data, and prepared the figures with assistance from Federica Sallusto. Anne Puel recruited participants and collected biological samples. Sinu Paul and Alessandro Sette performed the peptide bioinformatic analysis. Simone Becattini and Federica Sallusto analyzed the data and wrote the original draft with contributions from Salomé LeibundGut‐Landmann, Jean‐Laurent Casanova, Anne Puel, and Antonio Lanzavecchia. All authors provided inputs and critical revision of the manuscript.

## Conflicts of Interest

The authors declare no conflicts of interest.

## Supporting information




**Supporting File**: eji70065‐sup‐0001‐SuppMat.pdf

## Data Availability

The data supporting the findings of this study are available in the Supporting Information Material of this article.
