# Supplementary material for: Abundant Yet Aberrant T Helper Cell Responses to Candida albicans Underlie Mucosal Candidiasis in Humans and Mice
Source: Eur J Immunol. 2025 Oct 6;55(10):e70065. doi: 10.1002/eji.70065 (PMC12501408; doi:10.1002/eji.70065)
Supplement: Supplementary file 1 — Supporting File: eji70065‐sup‐0001‐SuppMat.pdf [file EJI-55-e70065-s001.pdf]

Abundant yet aberrant T helper cell responses to *Candida albicans* underlie mucosal candidiasis in humans and mice

Camilla Basso[1,#], Corinne De Gregorio[1,#], Roberta Marzi[1,#], Florian Kirchner[2], Gabor Gyülveszi[1], Mélanie Migaud[3,4], Sinu Paul[5], Alessandro Sette[5], Antonio Lanzavecchia[6], Salomé LeibundGut-Landmann[2,7], Jean-Laurent Casanova[3,4,8,9,10], Anne Puel[3,4,8], Simone Becattini[1,11,12,§], Federica Sallusto[1,13,§]

[1]Institute for Research in Biomedicine, Università della Svizzera italiana, Bellinzona, Switzerland; [2]Section of Immunology, Vetsuisse Faculty, and Institute of Experimental Medicine, University of Zürich, Zürich, Switzerland; [3]Laboratory of Human Genetics of Infectious Diseases, Necker Branch, INSERM U1163, Paris, France; [4]Paris Cité University, Imagine Institute, Paris, France; [5]La Jolla Institute for Immunology, La Jolla, USA; [6]National Institute of Molecular Genetic, Milan, Italy; [7]Medical Research Council Centre for Medical Mycology at the University of Exeter, Department of Biosciences, Faculty of Health and Life Sciences, Exeter, UK; [8]St. Giles Laboratory of Human Genetics of Infectious Diseases, Rockefeller Branch, Rockefeller University, New York, NY, USA; [9]Department of Pediatrics, Necker Hospital for Sick Children, Paris, France; [10]Howard Hughes Medical Institute, New York, NY, USA; [11]Department of Pathology and Immunology, University of Geneva, Switzerland; [12]Geneva Centre for Inflammation Research, University of Geneva, Switzerland; [13]Institute of Microbiology, ETH Zurich, Switzerland.

[#, §] Equal contributions

Correspondence: Federica Sallusto (federica.sallusto@irb.usi.ch) or Simone Becattini (Simone.Becattini@unige.ch)

Supplementary Table 1. Patients and controls included in the study.

Id	Family	Birth date (year)	Sex	Gene	Mutation	Reference
**P1**	A	2009	M	STAT1	M390I	K28 P36 (54)
**P2**	B	1998	F	STAT1	R274Q	K47 P68 (54)
**P3**	C	1981	M	STAT1	R274Q	K35 P46 (54)
**P4**	C (mother of P3)	1951	F	STAT1	R274Q	K35 P47 (54)
**P5**	D	1998	F	STAT1	A267V	K52 P77 (54)
**P6**	E	2005	M	STAT1	K344E	K51 P76 (54)
**P7**	F	2006	M	STAT1	K388E	K128 P202 (54)
**P8**	F (sister of P7)	2008	F	STAT1	K388E	K128 P203 (54)
**P9**	G	2005	M	STAT1	K344E	K51 P76 (54)
**P10**	H	1976	F	STAT1	M392T	K54 P79 (54)
**P11**	I	1972	F	STAT1	M202I	K45 P62 (54)
**P12**	I (son of P11)	2003	M	STAT1	M202I	K45 P64 (54)
**P13**	I (son of P11)	2007	M	STAT1	M202I	K45 P65 (54)
**Ctrl 1**	G (father of P9)	1972	M	STAT1	WT	–
**Ctrl 2**	H (mother of P10)	1937	F	STAT1	WT	–
**Ctrl 3**	–	n.a.	n.a.	STAT1	WT	–
**Ctrl 4**	–	n.a.	n.a.	STAT1	WT	–
**Ctrl 5**	–	n.a.	n.a.	STAT1	WT	–
**Ctrl 6**	–	n.a.	n.a.	STAT1	WT	–
**Ctrl 7**	–	n.a.	n.a.	STAT1	WT	–
**Ctrl 8**	–	n.a.	M	STAT1	WT	–
**Ctrl 9**	–	1970	M	STAT1	WT	–
**Ctrl 10**	–	1989	M	STAT1	WT	–

n.a., not available

Supplementary Table 2. Selected proteins for immunogenicity screening.

Pool n.	Protein	Uniprot	Peptides (n)	References
**1**	G3PDH	Q5ADM7	8	(59, 60)
**2**	FBA	Q9URB4	9	(60-62)
**3**	CCW14	Q5AFN8	4	(63, 64)
**4**	SAP10p	Q5A651	15	(65-68)
**5**	PLB4	Q59NM2	29	(68, 69)
**6**	SAP3p	P0CY29	16	(65-68)
**7**	SAP1p	P0CY27	14	(65-68)
**8**	SAP2p	P0DJ06	17	(65-68)
**9**	SAP5p	P43094	15	(65-68)
**10**	SAP4p	Q5A8N2	15	(65-68)
**11**	SAP6p	Q5AC08	17	(65-68)
**12**	LIP7	Q9P4E7	28	(70, 71)
**13**	PGA26	Q5AA09	5	(64, 72, 73)
**14**	SOD6	Q5ACV9	10	(64, 73-76)
**15**	PLB1	Q59W74	26	(68, 69)
**16**	CHT1	Q5AAH2	20	(64, 77)
**17**	ALS5	Q5A8L3	34	(66, 78, 79)
**18**	ALS6	Q5A2Z7	36	(66, 78, 79)
**19**	DCW1	Q5AD78	26	(80)
**20**	TOS1	Q5AJA4	13	(73, 75)
**21**	RBR1	Q5A6M0	5	(81, 82)
**22**	CRH11	Q5AFA2	12	(64, 83)
**23**	LIP2	Q5APG1	15	(70, 71)
**24**	LIP10	Q9P4E5	24	(70, 71)
**25**	LIP3	Q5APA9	21	(70, 71)
**26**	LIP6	Q9P4E8	15	(70, 71)
**27**	LIP1	Q5APE4	24	(70, 71)
**28**	ALS7	Q5A312	51	(66, 78, 79)
**29**	PLB5	Q5A760	23	(68, 69)
**30**	EAP1	G1UBC2	7	(84)
**31**	YWP1	Q59Y31	15	(77, 85)
**32**	HYR1	Q5AL03	21	(66, 86)
**33**	SOD5	Q5AD07	7	(64, 73-76)
**34**	SOD4	Q5AD05	6	(64, 73-76)
**35**	SIM1	Q5AKU5	6	(73, 77)
**36**	PIR1	Q59SC4	8	(64, 70)
**37**	RBT5	Q59UT4	6	(64)
**38**	PGA10	Q59UP6	7	(64, 72, 73)
**39**	MP65	Q59XS9	10	(87, 88)
**40**	LIP8	Q59PP4	27	(70, 71)
**41**	LIP4	Q59RI6	28	(70, 71)
**42**	LIP5	Q9P8W0	24	(70, 71)

**43**	LIP9	Q9P4E6	22	(70, 71)
**44**	PGA4	Q5AJY5	16	(64, 72, 73)
**45**	PHR2	O13318	22	(89, 90)
**46**	ALS4	Q59L09	14	(66, 78, 79)
**47**	ALS2	O74657	16	(66, 78, 79)
**48**	KEX2	Q5APK9	27	(91)
**49**	ALS9	Q5A8K7	15	(66, 78, 79)
**50**	CSA1	G1UB63	27	(63)
**51**	ALS1	Q5A8L0	22	(66, 78, 79)
**52**	ALS3	Q59L12	14	(66, 78, 79)
**53**	CHT2	P40953	14	(64, 77)
**54**	PLB2	Q59W34	26	(68, 69)
**55**	CHT3	P40954	15	(64, 77)
**56**	EXG2	Q5AIA1	13	(92)
**57**	PGA1	Q5ACL7	8	(64, 72, 73)
**58**	PLB4.5	Q5ALY4	13	(68, 69)
**59**	KRE1	P0CY22	9	(93, 94)
**60**	PRA1	P87020	11	(95)
**61**	ADH1	Q5AK23	11	(61)
**62**	SAP7	Q59VH7	20	(65-68)
**63**	RBT1	Q59TK9	20	(64)
**64**	HWP2	Q59PF9	15	(60, 66, 96)
**65**	SUN41	Q59NP5	5	(97)
**66**	UTR2	Q5AJC0	12	(90, 98, 99)
**67**	SAP9	Q59SU1	27	(65-68)
**68**	HWP1	P46593	6	(60, 66, 96)
**69**	IHD1	Q5A8I8	4	(100)
**70**	PGA13	Q5A343	11	(64, 72, 73)
**71**	CDC19	P46614	17	(61, 101)
**72**	PGK1	P46273	10	(60, 61, 101)
**73**	ENO1	P30575	15	(102)
**74**	HEX1	Q59NY2	26	(103)
**75**	MET6	P82610	28	(60, 61, 71)
**76**	SAP8	Q5AEM6	17	(65-68)
**77**	DFG5	Q5ACZ2	21	(80)
**78**	CRH12	Q5AK54	18	(64, 83)
**79**	PHR1	P43076	19	(89, 90)
**80**	RBR3	Q5A5F8	10	(81, 82)

Eighty *C. albicans* cytosolic, cell-wall associated or secreted proteins were selected (Uniprot accession code indicated) and predicted peptides from each sequence were produced ((number indicated) and pooled.

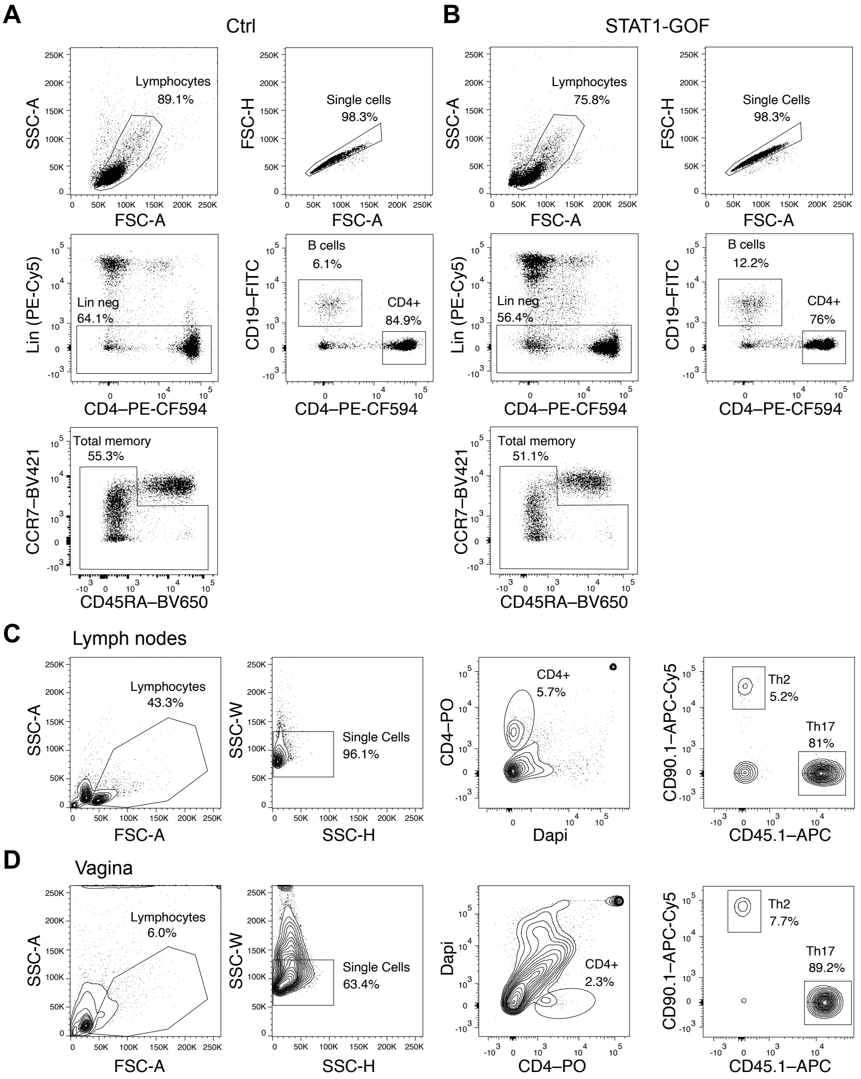

Supplementary Figure 1. Gating strategy of human and mouse T cells. A, B. Representative dot plots showing the gating strategy for human memory Th cell subsets in a healthy donor (Ctlr, A) and a STAT1-GOF patient (B). **C, D**. Representative dot plots showing the gating strategy for adoptively transferred Hector $CD4^+$ $CD90.1^+$ or $CD4^+$ $CD45.1^+$ T cells in draining lymph nodes (C) or vaginal tissues (D). PO=Pacific orange.

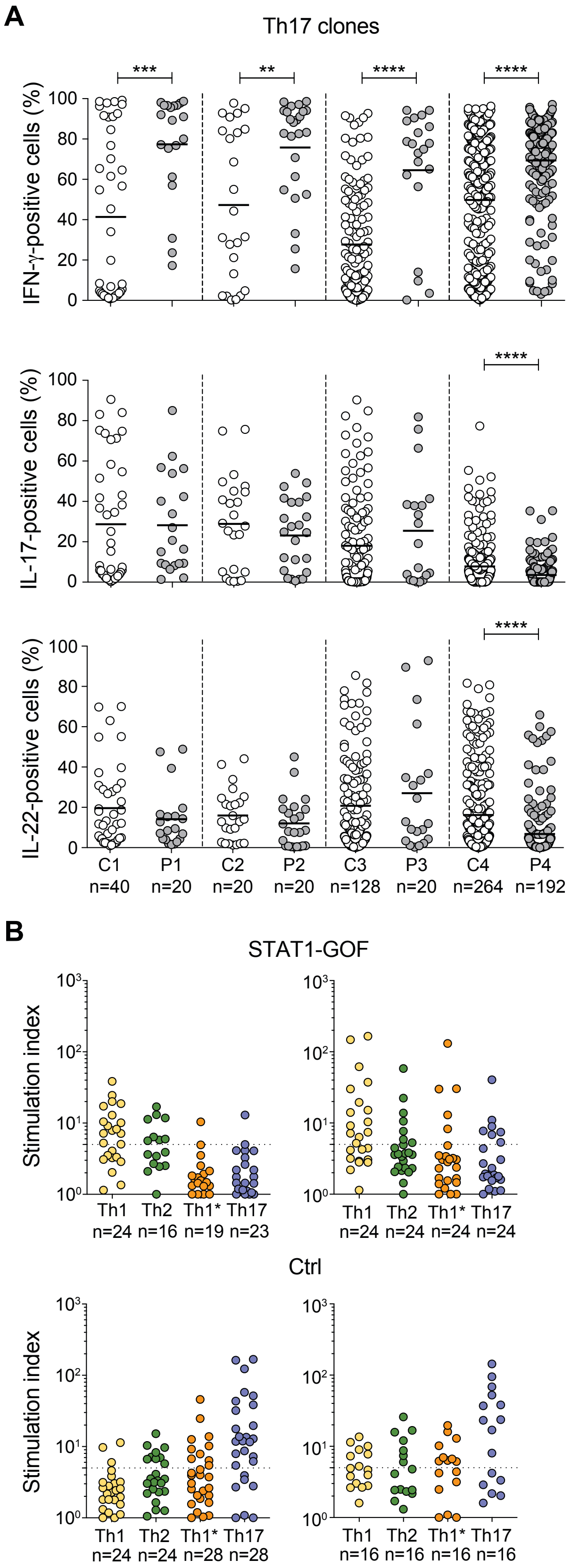

Supplementary Figure 2. A. T cell clones were obtained by limiting dilution from CCR6$^+$CXCR3$^-$ memory Th17 cells sorted from PBMCs of STAT1-GOF patients (P) and controls (C). Cytokine production was assessed by intracellular staining. Each dot represents

a T cell clone. Shown is the percentage of cytokine producing cells within individual T cell clones, n is the number of individual T cell clones tested, lines represent the mean. Significance analyzed by unpaired parametric *t*-test (with Welch's correction), $^{**}p<0.01$, $^{***}p<0.001$, $^{****}p<0.0001$. **B**. Representative data for T cell library screening. Memory Th1, Th2, Th1* and Th17 subsets from two STAT1-GOF patients and two healthy individuals (Ctrl) were polyclonally expanded in multiple wells. The number of wells ranged from 16 to 28 depending on the number of cells isolated and are indicated on the lower part of graphs. The individual T cell lines (each represented by a single dot) were screened for the response to autologous monocytes pulsed with *C. albicans*. Proliferation was assessed on day 4 after 16h-pulse with [^{3}H]-thymidine. Data are expressed as Stimulation Index (SI) = counts per minute with antigen / counts per minute without antigen. Dotted lines represent the cut-off value (positive ≥5).

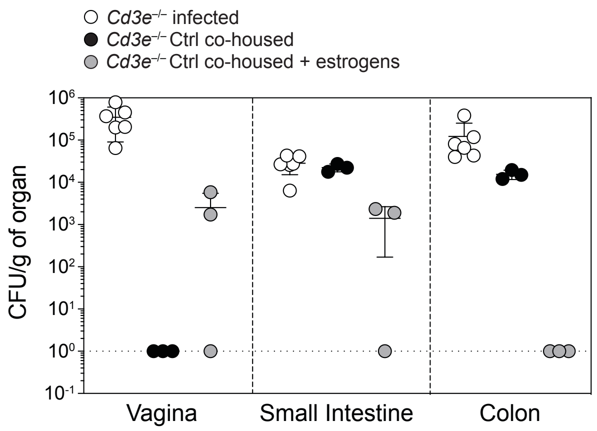

Supplementary Figure 3. *C. albicans*-infection spreading in co-housed animals. *C. albicans*-infected *Cd3e*$^{-/-}$ mice (n=5) were housed alone or co-housed for 50 days together with non-infected control *Cd3e*$^{-/-}$ mice that had (n=3) or had not (n=3) been pre-treated with estrogen. CFU count per gram of tissue. Shown are data from individual mice and mean ± s.d..

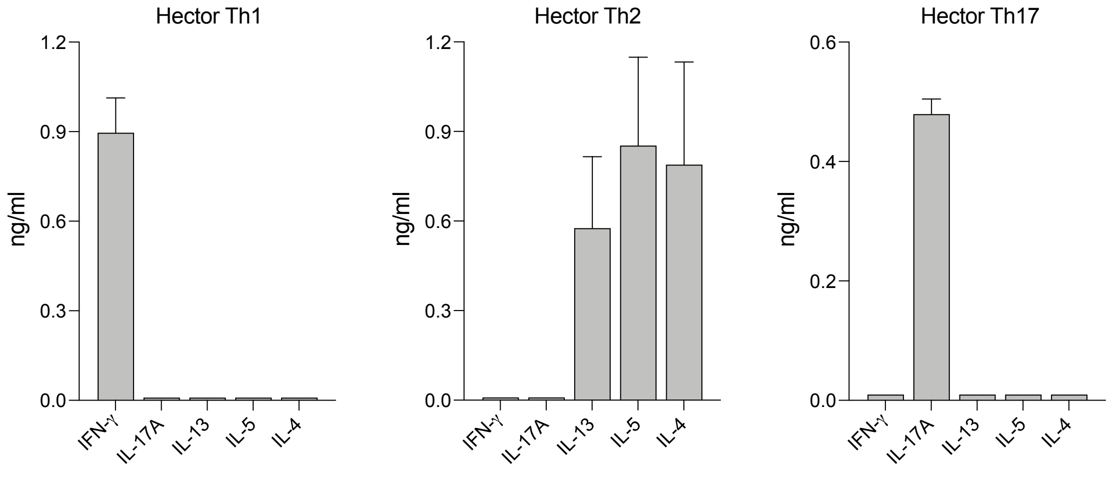

Supplementary Figure 4. Cytokine production by in vitro polarized Hector CD4$^+$ T cells. FACS-sorted Hector naïve CD4$^+$ T cells were primed *in vitro* in Th1-, Th2-, or Th17-polarizing conditions. Cytokines in cell culture supernatants were measured by ELISA on day 5. Bars represent mean + s.d. of at least 10 independent cultures per condition. One representative experiment of two performed.

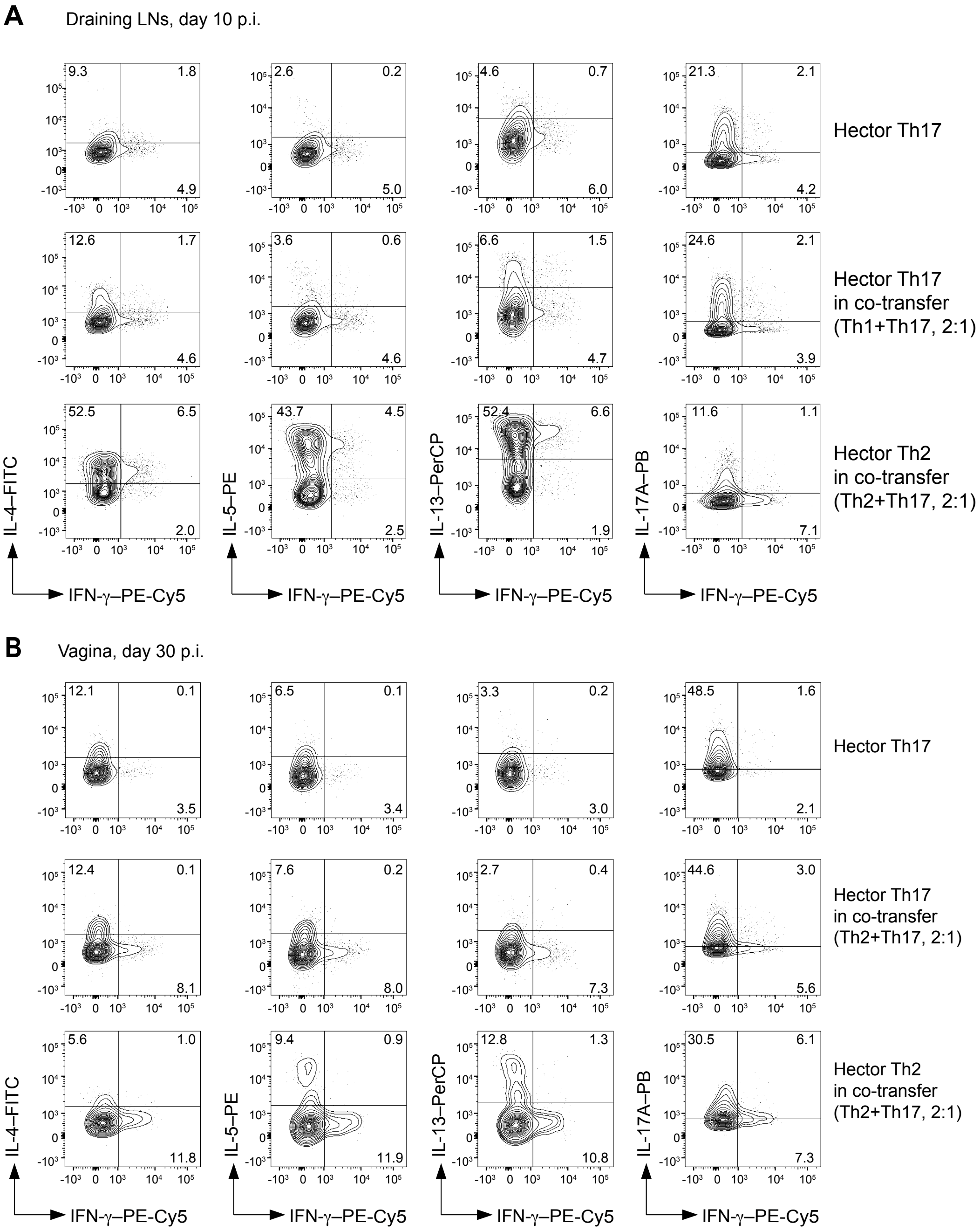

Supplementary Figure 5. Cytokine production by adoptively-transferred Hector Th2 and Th17 cells. *Cd3e*$^{-/-}$ mice were reconstituted on day -1 with in vitro polarized Hector Th17 alone or together with Hector Th2 cells (Th2-Th17 ratio = 2:1). The day after, mice were infected intravaginally with *C. albicans*. **A, B**. Intracellular cytokine staining of Hector Th cells recovered from dLNs on day 10 p.i. (A) or recovered from the vaginal tissue on day 30 p.i. (B). Cells were identified based on the expression of congenic markers. Recovered cells were restimulated *in vitro* for 5h with PMA and ionomycin in the presence of BFA for the last 2h before intracellular cytokine staining. PB=Pacific Blue.

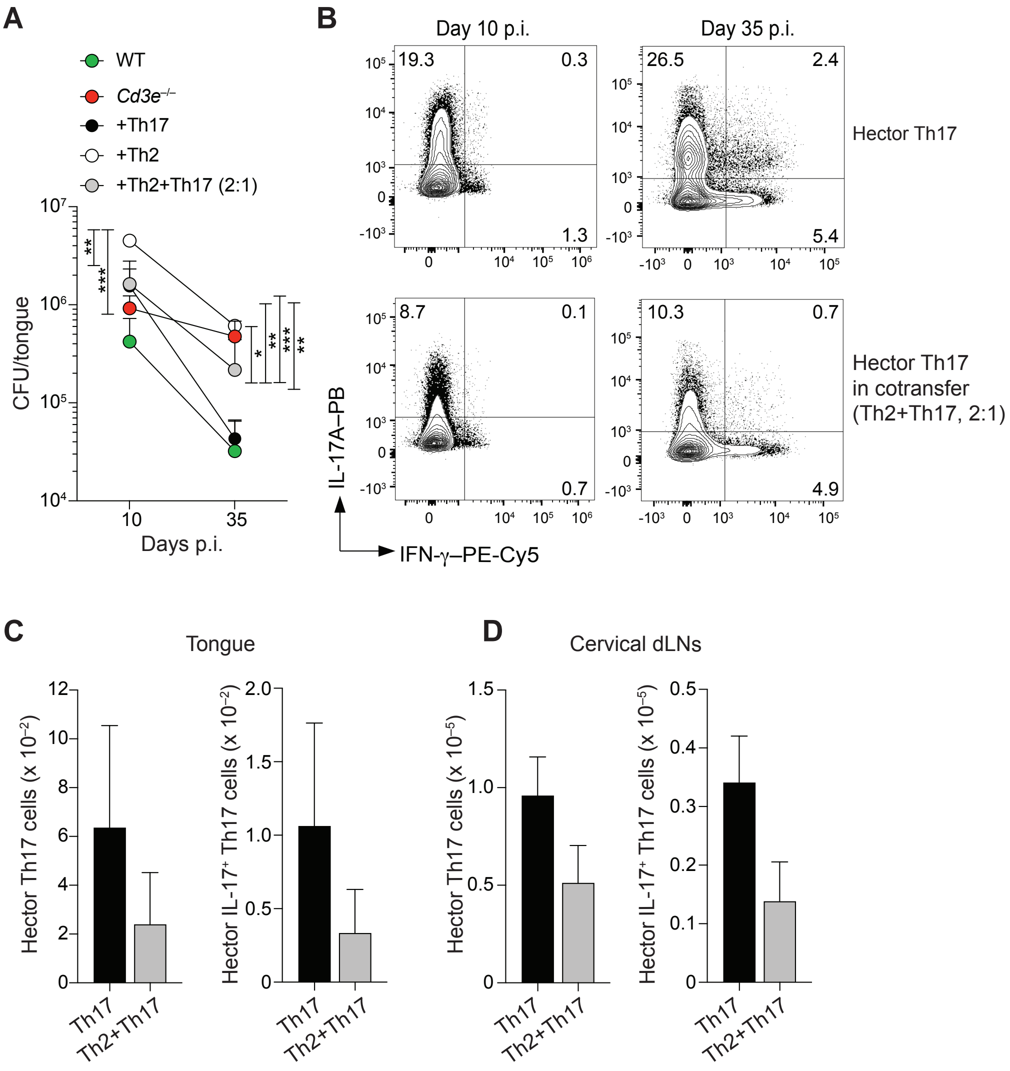

Supplementary Figure 6. Th2-inhibition of Th17-mediated protection in a model of oropharyngeal candidiasis. *Cd3e*$^{-/-}$ mice reconstituted on day -1 with in vitro polarized Hector $CD4^+$ T cells were infected with *C. albicans* sublingually. *Cd3e*$^{-/-}$ with no transferred T cells and WT C57BL/6 mice served as control. **A**. CFUs in the tongue of the indicated mouse groups (n=5 per each experimental group). Shown is the mean + s.d.. Statistical significance was determined by one-way Anova test. **B**. Representative FACS plot of cytokine production by Hector Th17 cells transferred in *Cd3e*$^{-/-}$ mice alone or in combination with Hector Th2 cells and analyzed on day 10 or day 35 p.i.. Cells were recovered from the cervical dLNs and then restimulated in vitro for 5 hrs with PMA and ionomycin in the presence of BFA for the last 2 hrs before intracellular cytokine staining. PB=Pacific Blue. **C, D**. Absolute numbers of Hector Th17 and Hector $IL\text{-}17A^+$ Th17 cells recovered from the tongue (n=5) (bars represent mean + s.d.) (**c**) and from the cervical dLNs (n=7) (bars represent the mean + s.e.m. pool of 2 independent experiments) (**D**) on day 35 p.i.. Statistical significance determined by Student's t test. **p*<0.05, ***p*<0.01, ****p*<0.001, *****p*<0.0001. Data are representative of two independent experiments.

References

59. Gil-Navarro I, Gil ML, Casanova M, O'Connor JE, Martinez JP, Gozalbo D. 1997. The glycolytic enzyme glyceraldehyde-3-phosphate dehydrogenase of Candida albicans is a surface antigen. *J Bacteriol* 179: 4992-9
60. Xin H, Dziadek S, Bundle DR, Cutler JE. 2008. Synthetic glycopeptide vaccines combining beta-mannan and peptide epitopes induce protection against candidiasis. *Proc Natl Acad Sci U S A* 105: 13526-31
61. Thomas DP, Viudes A, Monteagudo C, Lazzell AL, Saville SP, Lopez-Ribot JL. 2006. A proteomic-based approach for the identification of Candida albicans protein components present in a subunit vaccine that protects against disseminated candidiasis. *Proteomics* 6: 6033-41
62. Xin H, Cartmell J, Bailey JJ, Dziadek S, Bundle DR, Cutler JE. 2012. Self-adjuvanting glycopeptide conjugate vaccine against disseminated candidiasis. *PLoS One* 7: e35106
63. De Groot PW, Hellingwerf KJ, Klis FM. 2003. Genome-wide identification of fungal GPI proteins. *Yeast* 20: 781-96
64. de Groot PW, de Boer AD, Cunningham J, Dekker HL, de Jong L, Hellingwerf KJ, de Koster C, Klis FM. 2004. Proteomic analysis of Candida albicans cell walls reveals covalently bound carbohydrate-active enzymes and adhesins. *Eukaryot Cell* 3: 955-65
65. De Bernardis F, Amacker M, Arancia S, Sandini S, Gremion C, Zurbriggen R, Moser C, Cassone A. 2012. A virosomal vaccine against candidal vaginitis: immunogenicity, efficacy and safety profile in animal models. *Vaccine* 30: 4490-8
66. Lain A, Elguezabal N, Amutio E, Fernandez de Larrinoa I, Moragues MD, Ponton J. 2008. Use of recombinant antigens for the diagnosis of invasive candidiasis. *Clin Dev Immunol* 2008: 721950
67. Sandini S, La Valle R, Deaglio S, Malavasi F, Cassone A, De Bernardis F. 2011. A highly immunogenic recombinant and truncated protein of the secreted aspartic proteases family (rSap2t) of Candida albicans as a mucosal anticandidal vaccine. *FEMS Immunol Med Microbiol* 62: 215-24
68. Schofield DA, Westwater C, Warner T, Nicholas PJ, Paulling EE, Balish E. 2003. Hydrolytic gene expression during oroesophageal and gastric candidiasis in immunocompetent and immunodeficient gnotobiotic mice. *J Infect Dis* 188: 591-9
69. Theiss S, Ishdorj G, Brenot A, Kretschmar M, Lan CY, Nichterlein T, Hacker J, Nigam S, Agabian N, Kohler GA. 2006. Inactivation of the phospholipase B gene PLB5 in wild-type Candida albicans reduces cell-associated phospholipase A2 activity and attenuates virulence. *Int J Med Microbiol* 296: 405-20
70. Ene IV, Heilmann CJ, Sorgo AG, Walker LA, de Koster CG, Munro CA, Klis FM, Brown AJ. 2012. Carbon source-induced reprogramming of the cell wall proteome and secretome modulates the adherence and drug resistance of the fungal pathogen Candida albicans. *Proteomics* 12: 3164-79
71. Luo T, Kruger T, Knupfer U, Kasper L, Wielsch N, Hube B, Kortgen A, Bauer M, Giamarellos-Bourboulis EJ, Dimopoulos G, Brakhage AA, Kniemeyer O. 2016. Immunoproteomic analysis of antibody responses to extracellular proteins of Candida albicans revealing the importance of glycosylation for antigen recognition. *J Proteome Res* 15: 2394-406
72. Eckert SE, Heinz WJ, Zakikhany K, Thewes S, Haynes K, Hube B, Muhlschlegel FA. 2007. PGA4, a GAS homologue from Candida albicans, is up-regulated early in infection processes. *Fungal Genet Biol* 44: 368-77
73. Sosinska GJ, de Groot PWJ, Teixeira de Mattos MJ, Dekker HL, de Koster CG, Hellingwerf KJ, Klis FM. 2008. Hypoxic conditions and iron restriction affect the cell-wall proteome of Candida albicans grown under vagina-simulative conditions. *Microbiology (Reading)* 154: 510-20

74. Frohner IE, Bourgeois C, Yatsyk K, Majer O, Kuchler K. 2009. Candida albicans cell surface superoxide dismutases degrade host-derived reactive oxygen species to escape innate immune surveillance. *Mol Microbiol* 71: 240-52
75. Gil-Bona A, Parra-Giraldo CM, Hernaez ML, Reales-Calderon JA, Solis NV, Filler SG, Monteoliva L, Gil C. 2015. Candida albicans cell shaving uncovers new proteins involved in cell wall integrity, yeast to hypha transition, stress response and host-pathogen interaction. *J Proteomics* 127: 340-51
76. Martchenko M, Alarco AM, Harcus D, Whiteway M. 2004. Superoxide dismutases in Candida albicans: transcriptional regulation and functional characterization of the hyphal-induced SOD5 gene. *Mol Biol Cell* 15: 456-67
77. Sorgo AG, Heilmann CJ, Dekker HL, Brul S, de Koster CG, Klis FM. 2010. Mass spectrometric analysis of the secretome of Candida albicans. *Yeast* 27: 661-72
78. Bar E, Gladiator A, Bastidas S, Roschitzki B, Acha-Orbea H, Oxenius A, LeibundGut-Landmann S. 2012. A novel Th cell epitope of Candida albicans mediates protection from fungal infection. *J Immunol* 188: 5636-43
79. Spellberg BJ, Ibrahim AS, Avanesian V, Fu Y, Myers C, Phan QT, Filler SG, Yeaman MR, Edwards JE, Jr. 2006. Efficacy of the anti-Candida rAls3p-N or rAls1p-N vaccines against disseminated and mucosal candidiasis. *J Infect Dis* 194: 256-60
80. Spreghini E, Davis DA, Subaran R, Kim M, Mitchell AP. 2003. Roles of Candida albicans Dfg5p and Dcw1p cell surface proteins in growth and hypha formation. *Eukaryot Cell* 2: 746-55
81. Lotz H, Sohn K, Brunner H, Muhlschlegel FA, Rupp S. 2004. RBR1, a novel pH-regulated cell wall gene of Candida albicans, is repressed by RIM101 and activated by NRG1. *Eukaryot Cell* 3: 776-84
82. She X, Zhang L, Chen H, Calderone R, Li D. 2013. Cell surface changes in the Candida albicans mitochondrial mutant goa1Delta are associated with reduced recognition by innate immune cells. *Cell Microbiol* 15: 1572-84
83. Pardini G, De Groot PW, Coste AT, Karababa M, Klis FM, de Koster CG, Sanglard D. 2006. The CRH family coding for cell wall glycosylphosphatidylinositol proteins with a predicted transglycosidase domain affects cell wall organization and virulence of Candida albicans. *J Biol Chem* 281: 40399-411
84. Li F, Svarovsky MJ, Karlsson AJ, Wagner JP, Marchillo K, Oshel P, Andes D, Palecek SP. 2007. Eap1p, an adhesin that mediates Candida albicans biofilm formation in vitro and in vivo. *Eukaryot Cell* 6: 931-9
85. Granger BL, Flenniken ML, Davis DA, Mitchell AP, Cutler JE. 2005. Yeast wall protein 1 of Candida albicans. *Microbiology (Reading)* 151: 1631-44
86. Luo G, Ibrahim AS, Spellberg B, Nobile CJ, Mitchell AP, Fu Y. 2010. Candida albicans Hyr1p confers resistance to neutrophil killing and is a potential vaccine target. *J Infect Dis* 201: 1718-28
87. Gomez MJ, Maras B, Barca A, La Valle R, Barra D, Cassone A. 2000. Biochemical and immunological characterization of MP65, a major mannoprotein antigen of the opportunistic human pathogen Candida albicans. *Infect Immun* 68: 694-701
88. Nisini R, Romagnoli G, Gomez MJ, La Valle R, Torosantucci A, Mariotti S, Teloni R, Cassone A. 2001. Antigenic properties and processing requirements of 65-kilodalton mannoprotein, a major antigen target of anti-Candida human T-cell response, as disclosed by specific human T-cell clones. *Infect Immun* 69: 3728-36
89. Choi W, Yoo YJ, Kim M, Shin D, Jeon HB, Choi W. 2003. Identification of proteins highly expressed in the hyphae of Candida albicans by two-dimensional electrophoresis. *Yeast* 20: 1053-60
90. Heilmann CJ, Sorgo AG, Siliakus AR, Dekker HL, Brul S, de Koster CG, de Koning LJ, Klis FM. 2011. Hyphal induction in the human fungal pathogen Candida albicans reveals a characteristic wall protein profile. *Microbiology (Reading)* 157: 2297-307
91. Newport G, Kuo A, Flattery A, Gill C, Blake JJ, Kurtz MB, Abruzzo GK, Agabian N. 2003. Inactivation of Kex2p diminishes the virulence of Candida albicans. *J Biol Chem* 278: 1713-20

92. Lee SA, Wormsley S, Kamoun S, Lee AF, Joiner K, Wong B. 2003. An analysis of the Candida albicans genome database for soluble secreted proteins using computer-based prediction algorithms. *Yeast* 20: 595-610
93. Boone C, Sdicu A, Laroche M, Bussey H. 1991. Isolation from Candida albicans of a functional homolog of the Saccharomyces cerevisiae KRE1 gene, which is involved in cell wall beta-glucan synthesis. *J Bacteriol* 173: 6859-64
94. Chaffin WL. 2008. Candida albicans cell wall proteins. *Microbiol Mol Biol Rev* 72: 495-544
95. Luo S, Blom AM, Rupp S, Hipler UC, Hube B, Skerka C, Zipfel PF. 2011. The pH-regulated antigen 1 of Candida albicans binds the human complement inhibitor C4b-binding protein and mediates fungal complement evasion. *J Biol Chem* 286: 8021-9
96. Hayek P, Dib L, Yazbeck P, Beyrouthy B, Khalaf RA. 2010. Characterization of Hwp2, a Candida albicans putative GPI-anchored cell wall protein necessary for invasive growth. *Microbiol Res* 165: 250-8
97. Hiller E, Heine S, Brunner H, Rupp S. 2007. Candida albicans Sun41p, a putative glycosidase, is involved in morphogenesis, cell wall biogenesis, and biofilm formation. *Eukaryot Cell* 6: 2056-65
98. Alberti-Segui C, Morales AJ, Xing H, Kessler MM, Willins DA, Weinstock KG, Cottarel G, Fechtel K, Rogers B. 2004. Identification of potential cell-surface proteins in Candida albicans and investigation of the role of a putative cell-surface glycosidase in adhesion and virulence. *Yeast* 21: 285-302
99. Heilmann CJ, Sorgo AG, Mohammadi S, Sosinska GJ, de Koster CG, Brul S, de Koning LJ, Klis FM. 2013. Surface stress induces a conserved cell wall stress response in the pathogenic fungus Candida albicans. *Eukaryot Cell* 12: 254-64
100. Cabral V, Znaidi S, Walker LA, Martin-Yken H, Dague E, Legrand M, Lee K, Chauvel M, Firon A, Rossignol T, Richard ML, Munro CA, Bachellier-Bassi S, d'Enfert C. 2014. Targeted changes of the cell wall proteome influence Candida albicans ability to form single- and multi-strain biofilms. *PLoS Pathog* 10: e1004542
101. Cabezon V, Llama-Palacios A, Nombela C, Monteoliva L, Gil C. 2009. Analysis of Candida albicans plasma membrane proteome. *Proteomics* 9: 4770-86
102. Franklyn KM, Warmington JR, Ott AK, Ashman RB. 1990. An immunodominant antigen of Candida albicans shows homology to the enzyme enolase. *Immunol Cell Biol* 68 (Pt 3): 173-8
103. Ruhela D, Kamthan M, Saha P, Majumdar SS, Datta K, Abdin MZ, Datta A. 2015. In vivo role of Candida albicans beta-hexosaminidase (HEX1) in carbon scavenging. *Microbiologyopen* 4: 730-42
